# Systems biology meets stress ecology: linking molecular and organismal stress responses in *Daphnia magna*

**DOI:** 10.1186/gb-2008-9-2-r40

**Published:** 2008-02-21

**Authors:** Lars-Henrik Heckmann, Richard M Sibly, Richard Connon, Helen L Hooper, Thomas H Hutchinson, Steve J Maund, Christopher J Hill, Anthony Bouetard, Amanda Callaghan

**Affiliations:** 1 University of Reading, School of Biological Sciences, Environmental Biology, Philip Lyle Building, Reading, RG6 6BX, UK; 2 University of Aarhus, National Environmental Research Institute, Department of Terrestrial Ecology, Vejlsøvej, DK-8600, Silkeborg, Denmark; 3 University of California, School of Veterinary Medicine, Department of Anatomy, Physiology and Cell Biology, Davis, California 95616, USA; 4 AstraZeneca Global SHE, Brixham Environmental Laboratory, Devon, TQ5 8BA, UK; 5 Plymouth Marine Laboratory, Prospect Place, The Hoe, Plymouth, PL1 3DH, UK; 6 Syngenta Crop Protection AG, 4002 Basel, Switzerland

## Abstract

**Background:**

Ibuprofen and other nonsteroidal anti-inflammatory drugs have been designed to interrupt eicosanoid metabolism in mammals, but little is known of how they affect nontarget organisms. Here we report a systems biology study that simultaneously describes the transcriptomic and phenotypic stress responses of the model crustacean *Daphnia magna *after exposure to ibuprofen.

**Results:**

Our findings reveal intriguing similarities in the mode of action of ibuprofen between vertebrates and invertebrates, and they suggest that ibuprofen has a targeted impact on reproduction at the molecular, organismal, and population level in daphnids. Microarray expression and temporal real-time quantitative PCR profiles of key genes suggest early ibuprofen interruption of crustacean eicosanoid metabolism, which appears to disrupt signal transduction affecting juvenile hormone metabolism and oogenesis.

**Conclusion:**

Combining molecular and organismal stress responses provides a guide to possible chronic consequences of environmental stress for population health. This could improve current environmental risk assessment by providing an early indication of the need for higher tier testing. Our study demonstrates the advantages of a systems approach to stress ecology, in which *Daphnia *will probably play a major role.

## Abstract 2

A study of the transcriptomic and phenotypic stress responses of the model crustacean Daphnia magna following exposure to ibuprofen shows similarities in its mode of action between vertebrates and invertebrates.

## Background

Organismal stress responses have been studied for decades in ecology and ecotoxicology to establish the factors that limit species distributions and to investigate the effects of anthropogenic activities [1]. It was not until recently, however, that stress responses were investigated at the genomic level to illuminate underlying mechanisms [2,3]. Studying stress responses individually at just one level of biological organization yield little insight into how the organism deals with stress overall, but integration of responses at different levels promotes a holistic understanding of the whole system. Knowledge of the phenotypic consequences of stress as well as the genomic components (for instance, genes) that are induced or suppressed enables us to identify not only the mode of action (MOA) of the stressor but also which genomic components affect organismal growth, reproduction and survival, and thus populations. So, increased knowledge of the fundamental interactions between genome and phenotype should enable us to predict population stress responses better.

In genomic nonmodel organisms an overview of global transcriptomic responses may be achieved through using, for example, Gene Ontology (GO) [4] and the Kyoto Encyclopedia of Genes and Genomes (KEGG) [5]. KEGG in particular facilitates a pathway-driven approach, which - within a toxicogenomic context - allows identification of general molecular stress response as well as highlighting biochemical pathways that are associated with stressor-specific responses. Recently, in stress ecology, a number of *Daphnia magna *Straus [6-8] and other invertebrate [9] microarray reports have been published, but few of these have integrated transcriptome and phenotype to an extent that clarifies the link between these biological levels. This may partly be because many environmental and chemical stressors have a very complex MOA and ecophysiological impact [10], which diminishes the feasibility of linking molecular and organismal levels. We previously identified the nonsteroidal anti-inflammatory drug (NSAID) ibuprofen as having a targeted impact on reproduction in *D. magna *following chronic exposure [11], making ibuprofen a good model stressor for integrating genomic and higher level phenotypic stress responses. In mammals, ibuprofen and other NSAIDs operate as reversible competitive inhibitors of the enzyme cycloxygenase (COX), which is responsible for metabolism of arachidonic acid (AA), an n-6 fatty acid, to produce eicosanoids (for instance, prostaglandins). Eicosanoids act as autocrine or paracrine signallers (local hormones) and are important regulators of reproduction, ion flux, and immunity in both vertebrates and invertebrates [12].

*Daphnia *spp. (Crustacea: Cladocera) have emerged as leading model invertebrates in ecological genomics (hereafter referred to as 'ecogenomics'), especially with the recent progress that has been made in sequencing of the *Daphnia pulex *genome [13] and to a lesser extent *D. magna *[14]. *Daphnia *spp. have some clear advantages as ecogenomic models compared with other commonly studied invertebrates used in genomics, such as *Caenorhabditis elegans *and *Drosophila melanogaster*. It is a key genus in lentic ecosystems, making it ecologically relevant, and daphnids are widely used in population studies and environmental risk assessments. Although gene expression of organisms with sexual reproduction varies considerably among individuals of similar age [15], genetic variability should be low in *Daphnia *spp., which mainly reproduce asexually through parthenogenesis. These characteristics mean that *Daphnia *spp. are the only aquatic arthropods that can be considered to be ideal ecogenomic models (*sensu *Feder and Mitchell-Olds [16]), being supported by a large scientific community and several thousand publications. Thus, *Daphnia *spp. have great potential in the study of genetic and molecular interactions, particularly in combination with phenotypic responses, because of the feasibility of monitoring changes to life history traits [11].

Here we report a systems biology study that simultaneously describes the transcriptomic and phenotypic stress responses of *D. magna *to ibuprofen. To gain insight into the molecular MOA of ibuprofen, and its impact on population health, we conducted a microarray study in conjunction with a chronic population experiment to study effects on life history traits and population dynamics. The chronic study revealed a dramatic reduction in reproduction, resulting in population decline, at the highest concentration of ibuprofen (reported in detail by Heckmann and coworkers [11]). The combined microarray and population study was followed up here by temporal transcriptomic profiling of selected genes using real-time quantitative PCR (QPCR), plus a further chronic study aimed at investigating phenotypic responses related to reproduction such as embryogenesis, moulting, and male production. Using microarrays we identified several interlinked pathways and biological processes in response to acute ibuprofen exposure, such as eicosanoid metabolism, peroxisome proliferator-activated receptor (PPAR) signaling, and oogenesis. This could be further integrated with the observed phenotypic stress response after chronic ibuprofen exposure (reduced fecundity and early arrest of embryogenesis). Temporal transcriptomic profiles of key genes confirmed early inhibition by ibuprofen of crustacean eicosanoid metabolism (for instance, the gene encoding leukotriene B_4 _12-hydroxydehydrogenase [LTB_4_DH]), which appears to disrupt signal transduction, affecting the *Daphnia *endocrine system related to juvenile hormone metabolism and oogenesis.

Our approach shows strong links between acute transcriptomic and chronic phenotypic stress responses, and shows promise for predicting chronic consequences of environmental stress for population health based on insights from the molecular MOA of the stressor. The results also highlight similarities between the eicosanoid pathways of vertebrates and invertebrates, and add support to the possibility of using MOA to aid in test species selection for assessment of the environmental safety of chemicals [17].

## Results

The microarray experiment consisted of quadruplicates of a control and three concentrations of ibuprofen, namely 20, 40, and 80 mg/l. Neonate (<24 hours old) *D. magna *(310 individuals/replicate) were used to facilitate linkage of acute transcriptomic (24 hours) and chronic effects (14 days) at higher levels throughout the first important part of the daphnid life cycle (developing from neonate to adult). Following 24 hours of exposure, 300 individuals/replicate were preserved for microarray hybridizations (one hybridization per replicate), whereas the remaining ten individuals were left in the test vessels to monitor chronic organismal and populations effects for a total exposure of 14 days (for further details, see Materials and methods [below] and the report by Heckmann and coworkers [11]).

Our custom-made microarray contains 13,000+ cDNAs covering about 5,000 unique *D. magna *genes. A total of 272 cDNAs were significantly differentially expressed after 24 hours of exposure to ibuprofen (see Additional data file 1). Interestingly, there was a significant positive linear relationship (R^2 ^= 0.99, *P *< 0.05) between downregulated genes and increasing ibuprofen concentration, with 36%, 39%, and 47% of genes being suppressed at ibuprofen concentrations of 20, 40, and 80 mg/l, respectively. Thus, as ibuprofen stress increased, global gene expression appeared to be reduced, suggesting that nonessential processes were suppressed, perhaps in order to save energy [3].

Following sequence analysis, 183 cDNAs were annotated (89 cDNAs had nonsignificant matches; see Additional data file 1). Removal of redundant sequences (same annotation or belonging to the same DaphniaBase sequence contig [18]) resulted in a final gene list of 96 unique genes. About 45% of these genes were more than twofold differentially expressed at one or more of the ibuprofen concentrations compared with control (see Additional data file 2). This revealed an overall strong molecular response to the treatment, considering that the transcriptomic data were based on whole organism homogenates. Genes were assigned to functional categories using GO (50 genes) and KEGG (46 genes), as shown in Table 1.

**Table 1:** Functional categorization of *Daphnia magna *genes responding to acute ibuprofen exposure

Process	Functional category	Induced	Suppressed
1 Metabolism	1.1 Carbohydrate metabolism		
	Glycolysis/Gluconeogenesis (PATH:00010)	***Eno***	
	Fructose and mannose metabolism (PATH:00051)	***MANA***	
	Starch and sucrose metabolism (PATH:00500)	***AmyA***, ***GlyP***^a^	
	1.2 Energy metabolism		
	Oxidative phosphorylation (PATH:000190)	***atpB***^b^, ***CYTB***, ***RISP***	***ANT***, ***CO1***^c^, ***CO2***^c^, ***ND4***^d^
	1.3 Lipid metabolism	*GM2AP*	
	Glycerolipid metabolism (PATH:00561)	***Lip***	
	Eicosanoid metabolism (PATH:00590)	*Ltb4dh*	***GPX***^e^
	1.4 Amino acid metabolism		
	Cysteine metabolism (PATH:00272)	***Sult1C***	
	1.5 Metabolisms of co-factors and vitamins		
	Nicotinate and nicotinamide metabolism (PATH:000760)		***Nt5***^f^
	1.6 Polypeptide metabolism (proteolysis)	*BPTI*	*Spint*
	Peptidases	*CATL1*, *CPA2*, *Ctrb2*, *SC1*, *Try*	*ASTL*, *SP*
2 Genetic information processing	2.1 Transcription		*Ubn*, *H4*
	2.2 Translation	*DEADc*, *Sep15*	
	Ribosome (PATH:03010)	***16S rRNA***, ***28S rRNA***, ***RpL6***, ***RpL14***, ***RpL15***, ***RpL28***, ***RpL30***, ***RpL38***, ***RpS2***, ***RpS3A***, ***RpS4***, ***RpS12***, ***RpS20***, ***RpS25***, ***RpS30***	***RpL9***, ***RpL22***, ***RpL27***, ***RpS10***, ***RpS13***, ***RpS17***
	2.3 Chaperones and folding catalysts	*HSP20*, *UBQ*	*PDI*
3 Environmental information processing	3.1 Membrane transport	*Inx2*	
	3.2 Signal transduction	*Ptn*, *Reep5*	
	MAPK signaling pathway (PATH:04010)	***HSP70***^g^	
	Calcium signaling pathway (PATH:04020)	***VDAC2***	
	Wnt signaling pathway (PATH:04310)	***RhoA***^h^	***CTBP***^i^, ***Skp1***^j^
	3.3 Signaling molecules and interaction		***CNTN1***
	3.4 Sensory system	*A10*	
4 Cellular processes	4.1 Cell motility	*MRLC2*, *Tm1*	
	4.2 Cell communication (PATH:01430)	***Reln***^k^	***Act***^h^
	4.3 Endocrine system		
	PPAR signaling pathway (PATH:03320)	***FABP3***	***ACS***^l^
	Oogenesis (vitellogenesis and oocyte maturation) maturation)	*DmagVTG1*	*JHE*, *LPD_N*, *VMO1*
	Moulting	*Cht*^m^, *ChtBD2*, *Gasp*, *LPCP29*, *Peritrophin-A*	*cap-2*, *ChtBD4*, *CP7*, *DD5*, *PCP16.7*
	4.4 Immune system		*CLECT*, *CUB*, *GNBP*
	4.5 Inorganic ion transport and metabolism	*AT1A*, *CRIP*, *Fer*, *Fer1HCH*, *dmHb2*, *VGCa*	*dmHb1*, *Sfat*, *Znf_AN1*

Genes presented in bold are directly linked to a Kyoto Encyclopedia of Genes and Genomes pathway, whereas other genes are listed with their functional category or subcategory based on Gene Ontology (see Additional data file 2). ^a^*GlyP *is also associated with the insulin signaling pathway (PATH:04910). ^b^*atpB *is also associated with the type III secretion system (PATH:03070), flagellar assembly (PATH:02040), and epithelial cell signaling in *Helicobacter pylori *infection (PATH:05120). ^c^*CO1 *and *CO2 *are also associated with the vascular endothelial growth factor signaling pathway (PATH:04370). ^d^*ND4 *is also associated with ubiquinone biosynthesis (PATH:00130). ^e^*GPX *is also associated with glutathione metabolism (PATH:00480). ^f^*Nt5 *is also associated with purine metabolism (PATH:00230) and pyrimidine metabolism (PATH:00240). ^g^*HSP70 *is also associated with antigen processing and presentation (PATH:004612) related to the immune system. ^h^*Act *and *RhoA *are involved in eight and nine different pathways, respectively. Here they have been associated with one that is already represented, but because of this multi-alignment they have not been considered any further. ^i^*CTBP *is also associated with the notch signaling pathway (PATH:04330). ^j^*Skp1 *is also associated with the cell cycle (PATH:04110), ubiquitin-mediated proteolysis (PATH:04120), and transforming growth factor-β signaling pathway (PATH:04350), which all interlink through the Wnt signaling pathway. ^k^*Reln *is also associated with focal adhesion (PATH:04510) and extracellular matrix receptor interaction (PATH:04512). ^l^*ACS *is also associated with the adipocytokine signaling pathway (PATH:04920) and fatty acid metabolism (PATH:00071). ^m^Although related to aminosugars metabolism (PATH:00530), *Cht *has been categorized with moulting because of the key role of the encoded enzyme (chitinase) in this process among arthropods. Full gene names are available in Additional data file 1. PPAR, peroxisome proliferator-activated receptor.

### Global transcriptomic response to ibuprofen stress

Twenty-three ribosome encoding and translation-related genes were affected by ibuprofen, with the vast majority induced (Table 1 [section 2.2]). This differs from previous global general stress responses in, for example, budding yeast, in which translation-related genes were mainly downregulated after application of several types of stress (for example, heat shock and oxidative stress) [3]. However, in agreement with previous work on general stress responses [10], there were several indications of proteolysis and homeostatic insult (Table 1 [sections 1.6 and 4.5]). Data from the same microarray on *D. magna *of similar age that were exposed (24 hours) to cadmium (a fundamentally different stressor) [19] revealed a number of common transcriptomic stress responses when compared with ibuprofen-stressed daphnids. This included, for instance, induction of glycolytic, proteolytic, homeostatic, and heat shock protein genes, as well as interruption of several genes that are involved in oxidative phosphorylation (energy metabolism) and translation.

Stressor-specific responses were also apparent. Ibuprofen and other NSAIDs are known anti-inflammatory agents; we therefore expected responses in genes such as CLECT (encoding C-type lectin like) that are involved in the immune system (Table 1 [section 4.4]). More importantly, a number of genes associated with the mammalian MOA of ibuprofen, such as *Lip *(triacylglycerol lipase) and *Ltb4dh *(leukotriene B_4 _12-hydroxydehydrogenase), were significantly upregulated (Table 1 [section 1.3]), representing a highly specific response. The enzyme encoded by *Lip *has been shown to be important for releasing AA for eicosanoid metabolism in mammals [20], thus representing a key precursor step. *Ltb4dh *is directly associated with eicosanoid metabolism, comprising one of the downstream steps of the lipoxygenase (LOX) pathway [12]. Although *Ltb4dh *responded on the microarray, the fluorescent emission levels were below the set detection criteria. This was perhaps an artefact of studying whole organism homogenates that inevitably dilute tissue-specific expression, because *Ltb4dh *is known to be induced in a concentration-dependent manner [21].

One of the most markedly suppressed genes, *JHE *(juvenile hormone esterase), plays an important role in vitellogenesis (yolk formation), which comprises an important part of invertebrate oogenesis [22] (Table 1 [section 4.3]). The encoded enzyme is a key regulator of insect juvenile hormone (JH) [23], and the equivalent crustacean JH, methyl farnesoate, is known to regulate daphnid vitellogenesis by suppressing expression of *DmagVTG1 *(vitellogenin 1) expression through binding to upstream JH-responsive elements [22]. Our microarray data did not confirm suppression of *DmagVTG1 *after 24 hours of exposure to ibuprofen (Table 1 [section 4.3]); they rather indicated upregulation (see Additional data file 2), but this may be a matter of timing (see Genes related to eicosanoid metabolism show early response to ibuprofen [below]). LeBlanc and colleagues [24] reported that JH co-regulates the production of both hemoglobin and male offspring in *D. magna*; thus, *dmHb2*, containing a JH-responsive element in its promoter region, is strongly upregulated by JH and JH analogs (JHAs) [25]. Ibuprofen induced *dmHb2 *at low effect concentrations in the present study (Table 1 [section 4.5]), but there was no phenotypic evidence of either increased production of hemoglobin (daphnids becoming distinctly red) or male offspring (see results presented below).

### Real-time quantitative PCR validation of microarray data

Six genes - *CLECT*, *DmagVTG1*, *GPX *(glutathione peroxidase), *JHE*, *Lip *and *Ubn *(ubinuclein) - covering different GOs were selected to validate the global expression profile (see Additional data file 3). Expression levels of the selected microarray responding genes were compared with QPCR results from *D. magna *exposed in a comparable independent experiment. Individual R^2 ^values ranged between 0.87 and 1.00 for the tested genes except *Ubn*, for which the R^2 ^value was 0.56 (see Additional data file 3). Overall, these QPCR responses validate the use of our microarray data.

### Genes related to eicosanoid metabolism show early response to ibuprofen

After microarray analysis of global transcriptional responses to ibuprofen, we conducted a temporal expression profile (2 to 48 hours) experiment on neonate (<24 hours old) *D. magna *(50 individuals/replicate) in order to investigate further the expression of key genes using QPCR. The treatments (control and 80 mg/l ibuprofen) were replicated four times for every time point (2, 4, 8, 24, and 48 hours) and ten genes were analyzed (for further details, see Materials and methods [below]). Four linked to eicosanoid metabolism (*Lip*, *Ltb4dh*, *CTP *[choline-phosphate cytidylyltransferase], and *COX*), and six genes were associated with signal transduction and endocrine functions (*Cht *[chitinase], *DmagVTG1*, *FABP3 *[fatty acid binding protein 3], *JHE*, *RXR *[retinoid × receptor], and *VMO1 *[vitelline outer layer membrane protein 1]). *COX *was included to clarify the interruption of eicosanoid metabolisms because it represents a key component of the MOA of ibuprofen in mammals. *RXR *was included because recently reported evidence shows that JHAs can change the expression of this receptor in *D. magna *[26]. *CTP *was employed as a 'negative control', because this gene is involved in a part of the glycerophospholipid metabolism that is less relevant to eicosanoid metabolism.

**Figure 1:**
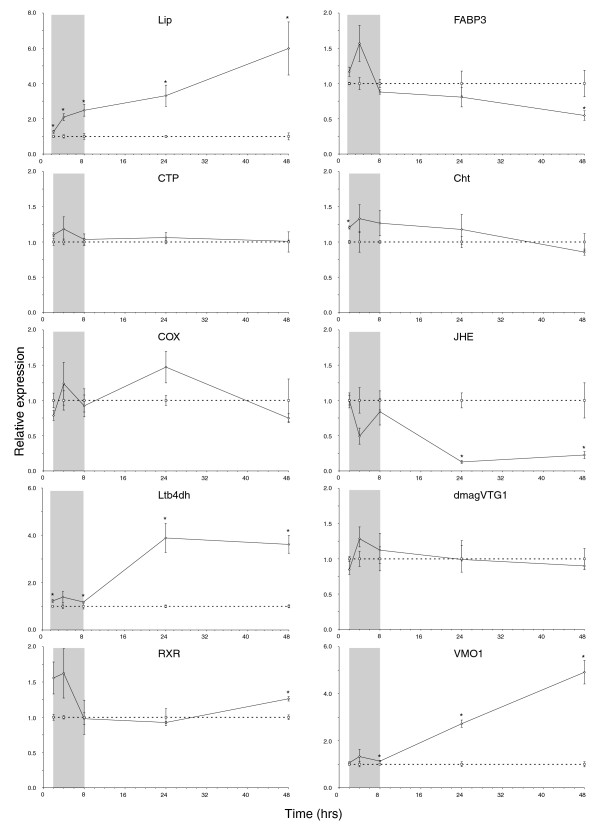
Temporal expression profiles of *Daphnia magna *genes after ibuprofen exposure. Shown are the temporal expression profiles of *D. magna *(<24 hours old) genes after 2 to 48 hours of exposure to ibuprofen (mean ± standard error). Gene expression was measured using quantitative PCR. Dotted and solid lines represent control and exposed (80 mg/l ibuprofen) expression, respectively. Target gene expression was calculated using DART-PCR [59] and normalized to a geNorm [60] estimated normalization factor based on the geometric mean of *Act *(actin), *GAPDH *(glyceraldehyde-3-phosphate dehydrogenase) and *UBC *(ubiquitin conjugating enzyme). Exposed expression levels are shown relative to controls at corresponding duration of exposure (note the different y-axes). The temporal 'gray zones' generally reveal fluctuating expression patterns perhaps reflecting homeostatic instability. Asterisks (*) denote a significant (*P *< 0.05, Student's *t*-test) difference from controls. The *Lip *(which encodes triacylglycerol lipase), *CTP *(choline-phosphate cytidylyltransferase), *Ltb4dh *(leukotriene B_4 _12-hydroxydehydrogenase) and *COX *(cycloxygenase) genes are related to lipid metabolism, whereas *RXR *(retinoid × receptor), *JHE *(juvenile hormone esterase), *DmagVTG1 *(vitellogenin 1), *VMO1 *(vitelline outer layer membrane protein 1), and *Cht *(chitinase) are associated with signal transduction and endocrine functions.

With the exception of *Lip*, the temporal expression of all of the analyzed genes fluctuated during early exposure (2 to 8 hours) to ibuprofen (Figure 1). We suggest that this fluctuation reflects a general homeostatic response. This could be an after effect of handling stress, but it may also show that daphnids are attempting to regulate toxicity during early stages of exposure. This early variation disappears by the classic ecotoxicological exposure time points of 24 and 48 hours, emphasizing the feasibility and importance of applying the latter.

The earliest genes to change expression levels significantly were the eicosanoid-related genes *Lip *and *Ltb4dh *(2 hours onward), with *Lip *being consistently upregulated throughout the exposure (Figure 1). As expected, the expression of the 'negative control' *CTP *was unchanged compared with controls. However, the temporal expression of *COX *was not significantly different from that of the controls, although there was a near significant (*P *= 0.088) upregulation after 24 hours of exposure, which may reflect COX inhibition (Figure 1).

**Figure 2:**
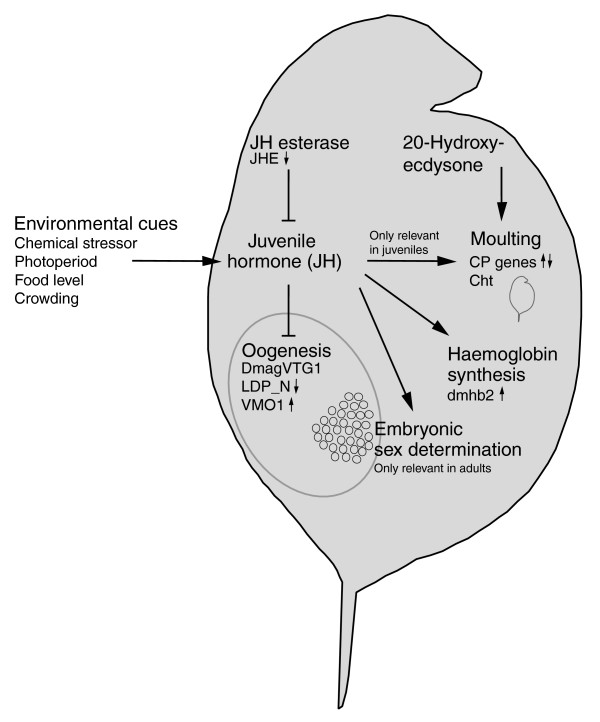
Overview of potential biological interactions of JH (methyl farnesoate) in *Daphnia magna*. Expression of relevant target genes in ibuprofen-stressed daphnids (24 to 48 hours of exposure) is indicated by small arrows quantified by either microarrays (normal font) or quantitative PCR (bold font). Note that 20-hydroxyecdysone is the main hormone controlling moulting in *Daphnia*, whereas juvenile hormone (JH; in arthropods) prevents sexual maturation between moults in juveniles. (There is currently no evidence from daphnids on this role.) Large arrows signify synthesis or induction of the particular product or process, whereas end bars denote inhibition. Abbreviations are as in Additional data file 1 and Figure 1; *CP *genes signify cuticle protein genes (see text for further details).

Global gene expression data showed that *Cht*, encoding a key moulting fluid enzyme secreted during apolysis [27], and several cuticle-related genes responded differentially to ibuprofen stress after 24 hours of exposure (Table 1 [section 4.3]). However, the temporal expression profile revealed that *Cht *was only significantly induced at 2 hours of exposure, after which there was no difference in expression between exposed and control daphnids (Figure 1). In arthropods, JH is involved in regulating both moulting (of sexually immature instars) and vitellogenesis [23], but there was no strong evidence that ibuprofen (or indirectly JH) affected moulting in the present study based on the temporal expression of *Cht *(Figure 1) and phenotypic results (see below). Figure 2 provides an overview of the potential biological interactions of JH (methyl farnesoate) in *D. magna *and related genes responding to ibuprofen stress. Evidence of elevated JH levels was strongly supported by the temporal suppression of *JHE *and late induction of *RXR *(Figure 1), suggesting that JH levels increase in exposed *D. magna *over time. JHA pyriproxyfen has been shown to suppress *DmagVTG1 *expression in 1-hour-old neonates after a 96-hour exposure [22]. However, a 48-hour exposure was too short to show a similar suppression of *DmagVTG1 *in older neonates (24 hours old), although there was a nonsignificant tendency toward suppression (Figure 1). *D. magna *start to ovulate (release mature oocytes into the brood chamber) when they are 5 to 6 days old at 20°C (Heckmann L-H, personal observations). Transcriptomic changes in vitellogenesis may therefore not be noticeable or relevant before daphnids become adolescent. Thus, it is likely that a decrease in *DmagVTG1 *expression would have been observed in the exposed 24-hour-old neonates if the temporal expression profile had been extended beyond 72 hours.

The microarray findings revealed that *VMO1 *was suppressed at 80 mg/l ibuprofen after 24 hours of exposure. However, the temporal expression of *VMO1 *was strongly upregulated after 24 hours of exposure and onward (Figure 1). In crustaceans, VMO1 proteins are synthesized outside the ovaries and are then transported via the hemolymph to developing oocytes. The major role of the vitelline membrane is to avoid mixing of yolk and albumen [28]. Expression of *VMO1 *seems to precede *DmagVTG1*, possibly revealing important functional insights into the timing of *D. magna *oogenesis.

### Ibuprofen reduces fecundity and arrests early embryogenesis

Although previous studies [11,29] showed that ibuprofen concentrations of 20 mg/l or greater suppress reproduction, questions remain as to whether ibuprofen acts on oogenesis or embryogenesis before hatching. A chronic experiment (8 days) was therefore conducted on adult 14-day-old *D. magna *(one individual/replicate) with five replicates of a control and three concentrations of ibuprofen, namely 20, 40 and 80 mg/l (for further details, see Materials and methods [below]). As expected exposure to more than 20 mg/l ibuprofen reduced fecundity, but it did not delay brood release or affect associated moulting (Table 2). The broods released after exposure to 80 mg/l ibuprofen had few viable neonates and consisted almost entirely of under-developed embryos (Table 2). Microscopic investigation showed that embryogenesis was arrested before completion of the first third of embryonic development - stage 2 *sensu *Kast-Hutcheson and coworkers [30] (see Additional data file 4).

**Table 2:** Reproduction of 14-day-old *Daphnia magna *exposed for 8 days to ibuprofen

	Brood	Control	20 mg/l ibuprofen	40 mg/l ibuprofen	80 mg/l ibuprofen
Brood release and adult moulting^a ^(age [days])	3rd	15.0 ± 0.00	15.2 ± 0.20	15.2 ± 0.20	15.2 ± 0.20
	4th	18.2 ± 0.20	18.4 ± 0.24	18.6 ± 0.24	18.4 ± 0.24
	5th	21.2 ± 0.20	21.4 ± 0.24	21.4 ± 0.24	21.2 ± 0.20
Fecundity (average number of offspring/brood)	3rd	22.8 ± 1.59	19.6 ± 2.42	18.8 ± 2.82	24.6 ± 3.14
	4th	28.4 ± 1.40	30.0 ± 0.95	8.20 ± 1.43*	0.00 ± 0.00*
	5th	42.0 ± 1.64	41.4 ± 2.25	6.60 ± 1.54*	1.00 ± 0.45*
Embryo abortion (average number of abortions/brood)	3rd	Nil	Nil	Nil	Nil
	4th	Nil	Nil	Observed^b^	Observed^b^
	5th	0.20 ± 0.20	0.20 ± 0.20	7.20 ± 3.20	20.6 ± 7.08*
Production of male offspring^c^	3rd	Na	NA	NA	NA
	4th	Nil	Nil	Nil	Nil
	5th	Nil	Nil	Nil	Nil

Values are expressed as mean ± standard error (*n *= 5). Asterisks (*) denote a significant (*P *< 0.05, analysis of variance) difference from controls. ^a^Moulting follows brood release in mature female daphnids and was assessed by counting shed carapaces (after moulting new eggs are deposited from the ovaries into the brood chamber with the incubation period corresponding to the intermoult period). ^b^Aborted embryos were not quantified, but a few were observed in 40 mg/l ibuprofen, and considerable numbers in 80 mg/l ibuprofen, comparable to 5^th ^brood. ^c^Fourth and fifth brood neonates were reared to adulthood (about 9 days) in uncontaminated media until every individual had produced their first brood. NA, not applicable.

Minor differences between the results presented here and those of our previous chronic studies, using adolescent [29] and neonate [11] individuals exposed for 10 and 14 days, respectively, suggest an ontogenetic shift in ibuprofen stress response, with fecundity being less affected in older individuals. This implies that susceptibility to the stressor (ibuprofen) decreases with age/size, which appears to be a common phenomenon in ecotoxicology [31].

Continued culturing of fourth and fifth brood neonates to adulthood in uncontaminated media did not reveal any induction of male offspring as a result of maternal exposure (Table 2). Comparing the number of fifth brood offspring, produced by adults of the control and 20 mg/l ibuprofen treatments, with the number of embryos aborted at 80 mg/l ibuprofen showed that on average there were 20 eggs fewer in the highest ibuprofen treatment (Table 2). This response was also observed in previous studies [11]. Fewer viable oocytes may have been deposited during ovulation possibly because of impaired ovarian maturation; while under-developed oocytes may have been re-absorbed by the mothers, a response was also found in stressed *D. melanogaster *following starvation [9]. Generally, it appears that ibuprofen primarily affects oogenesis and that embryogenesis in viable oocytes is arrested at high concentrations.

### A putative molecular mode of action of ibuprofen in *Daphnia *spp

**Figure 3:**
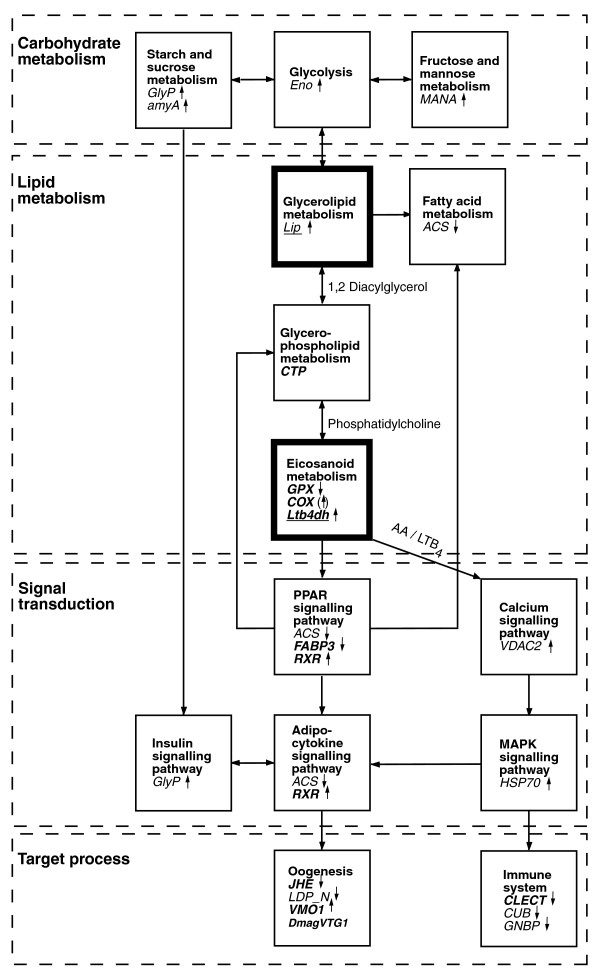
Pathways affected in *Daphnia magna *after acute ibuprofen exposure. Presented is a simplified overview of pathways affected in *D. magna *following acute exposure to ibuprofen, showing a network of 21 interlinked genes based on Kyoto Encyclopedia of Genes and Genomes representing a stress-specific response. Expression of target genes is indicated by small arrows quantified by either microarrays after 24 hours of exposure (normal font) or quantitative PCR temporal expression based on 24 to 48 hours of exposure (bold font). *GPX *and *CLECT *are based on quantitative PCR validation data. Bold boxes and underlined genes represent the initiation point of the ibuprofen mode of action (see Figure 1). Abbreviations are as in Additional data file 1 and Figure 1 (see text for further details).

Based on our microarray (Table 1) and temporal QPCR expression data (Figure 1), we constructed a diagram showing how the genes responding to ibuprofen exposure in *D. magna *can be tied into a pathway that links the putative molecular MOA of ibuprofen with carbohydrate metabolism, lipid metabolism, signal transduction, and two main biological target processes, namely oogenesis and the immune system (Figure 3). Our experimental design was not intended to allow study of phenotypic immune responses, even though eicosanoids play a vital role in invertebrate immune systems [12]. However, future studies may clarify whether ibuprofen-stressed crustaceans are more susceptible to infections because of the apparent repression of their immune system.

The temporal expression data indicated that lipid metabolism was initially affected (for example, *Lip *and *Ltb4dh*), with subsequent effects on carbohydrate metabolism and signal transduction ultimately affecting oogenesis (Figure 3); the latter was likewise evident from our phenotypic experiments (Table 2). The obvious genetic link between *Lip *(glycerolipid metabolism) and *Ltb4dh *(eicosanoid metabolism) would be *PLA2 *(phospholipase A_2_), which encodes a key enzyme that is responsible for hydrolyzing phosphatidylcholine into AA (Figure 3), comprising one of the first steps in eicosanoid metabolism [12]. Unfortunately, the DNA sequence of *PLA2 *was not available to us, but future studies should aim to identify this key gene in *D. magna*.

## Discussion

We found a strong link between transcriptomic and phenotypic stress responses in *D. magna *by integrating data on the molecular MOA of ibuprofen with ecophysiological effects observed at higher biological levels. Furthermore, to our knowledge, this is among the first studies to investigate the global transcriptomic stress response of an invertebrate exposed to a NSAID. Previous findings in *Bacillus megaterium *[32] suggest that NSAIDs mimic endogenous fatty acids and may interact with transcriptional regulation of eicosanoid target genes.

The transcription of *COX*, the target pathway in mammalian models, was only weakly affected by ibuprofen, restricting possible conclusions regarding whether ibuprofen is a COX inhibitor in daphnids until more (proteomic) evidence is available. However, this could suggest that ibuprofen has a LOX rather than a COX based MOA in crustaceans, because *Ltb4dh *was responding. Alternatively, it may reflect ontogenetic differences, whereby the COX pathway is less important or not activated in neonates. This idea is supported by differences in fecundity between organisms exposed as either neonates or adults. Individuals exposed as adults had a higher fecundity than those exposed as neonates [11,29]. Adult daphnids may have a relatively higher content of eicosanoids (and phospholipids) in the ovaries that may increase their tolerance by buffering the impact of ibuprofen stress on eicosanoid metabolism. Nevertheless, *Lip *expression was increased in ibuprofen-stressed neonates, indicating potential recruitment of AA [20] that may buffer the competitive inhibition of COX or LOX by increasing substrate availability. However, increased release of AA could affect signal transduction through the calcium signaling pathway (Figure 3), because AA has been shown to be involved in embryonic calcium signaling [33].

In mammals, prostaglandins and leukotrienes act as ligands at distinct transmembrane G-protein-coupled receptors and nuclear PPARs [34]. PPARs are transcription factors that form heterodimers with retinoid × receptor (upregulated in this study) and bind to target genes involved in, for instance, controlling prenatal and postnatal development [35,36]. Retinoid × receptor (encoded by *RXR*) also forms heterodimer complexes with other nuclear receptors and is known to bind JH in *D. magna *[26]. It remains unknown whether JH and eicosanoids interact directly in daphnids or whether the upregulation of *RXR *relates to PPAR and thus eicosanoid metabolism, rather than being involved with JH. The PPAR-α pathway is activated by leukotriene (LT)B_4 _in mammals [34], indicating that this could constitute the main signal transduction cut-off in ibuprofen-stressed *D. magna*. This is further supported by ibuprofen suppression of other PPAR-related genes, such as *ACS *and *FABP3 *(Figure 3); the latter of these two genes encodes fatty acid binding protein 3, which is involved in transporting PPAR ligands to the nucleus [35]. The LTB_4_DH enzyme, encoded by *Ltb4dh*, inactivates LTB_4_, and also catalyzes the degradation of the prostaglandin (PG)E_2 _and PGF_2α _[37]. Mammalian *in vitro *research has demonstrated that LTB_4_DH activity is strongly suppressed by the NSAIDs diclofenac and indomethacin, whereas ibuprofen has only a moderate effect [38]. Thus, induced expression of *Ltb4dh *in ibuprofen-stressed daphnids could reflect inhibition of LTB4DH activity, which may affect the catabolism of relevant eicosanoids. In relation to the reduced fecundity observed in this study, LTB_4 _has been shown to play an important role in yolk uptake during oogenesis in insects [39], as well as being an agonist for calcium signaling regulating mitosis in echinoderm eggs and embryos [40].

Decreased fecundity and induction of male offspring have been identified when exposing *D. magna *to JH and JHAs [41]. We have revealed a concentration-dependent reduction in fecundity after ibuprofen exposure [11], but the chronic follow-up experiment indicated that ibuprofen did not result in production of male offspring. Our transcriptomic data (*JHE *and *RXR*) strongly indicate that there was a higher than normal presence of JH, but why were no male offspring being produced in response to elevated JH levels? In many studies on daphnid and crustacean endocrinology (for review, LeBlanc [42]) individuals are exposed to high concentrations of potent JHAs, thus potentiating the normal JH signal that may lead to male induction in daphnids. If JH levels are elevated in ibuprofen-stressed daphnids, then we propose that the endocrine signal produced by JH was sufficient to reduce fecundity but insufficient to initiate production of male offspring. This suggests that JH signal transduction is tiered, firstly initiating a reduction in fecundity and secondly - if the signal is maintained or increased - causing ontogenetic sex change among the embryos. This type of signal transduction is robust and would make ecological sense. *Daphnia *mainly reproduce through cyclic parthenogenesis, but males are produced after strong environmental cues (for instance, fading light levels causing algae production to cease) to allow sexual reproduction and the formation of diapausing eggs (ephippia).

In summary, based on our genetic and phenotypic data, we suggest that the MOA of ibuprofen in *D. magna *functions as follows. Initially, ibuprofen interrupts eicosanoid metabolism, which is evident from the early response of *Lip *and *Ltb4dh*. This impairs normal signal transduction possibly through the PPAR and/or the calcium signaling pathway, which leads to disruption of the endocrine system related to JH metabolism (*JHE *and *RXR*) and oogenesis (*DmagVTG1 *and *VMO1*; Figure 3). The phenotypic response links strongly with reproduction showing reduced fecundity. We assume that fecundity is affected by disruption of normal JH metabolism caused by elevated JH levels in ibuprofen-stressed daphnids, or alternatively that ibuprofen mimicks JH, which halts vitellogenesis and thus oogenesis. Suppressed vitellogenesis (*DmagVTG1*) and under-development of the vitelline membrane (*VMO1*) would result in poor accumulation of protein, lipids, and other nutrients in the oocytes, as well as incomplete division of yolk and albumen. This would lead to failing oogenesis, with abnormal oocytes possibly being re-absorbed [9], or eventually arrested embryogenesis caused by nutrient deficiency. Ye and coworkers [43] showed that downregulation of COX-2 reduced the levels of PGE_2 _and PGI_2_, leading to delayed development and death of mice embryos. PGE_2 _has likewise been shown to play a key role during crustacean reproduction (vitellogenesis), possibly controlling ovulation [44,45]. Future proteomic investigations of LTB_4 _and PGE_2 _may further elucidate the role of these eicosanoids in daphnid reproduction. Furthermore, a recent study conducted in queen bees [46] showed that JH affects the expression of *vitellogenin *and insulin/insulin-like growth factor-1 signaling genes in opposite directions. In the present study, we found indications of repressed expression of *DmagVTG1 *coinciding with a consistent upregulation of genes that are involved in carbohydrate metabolism, and especially glycolysis, which is closely related to insulin signaling in both vertebrates and invertebrates [47] (Figure 3). However, the link between insulin, vitellogenin and JH, and the consequence of this interaction for daphnid reproduction remain to be unraveled.

## Conclusion

Our systems biology approach to stress ecology has proved fruitful in linking transcriptomic data with ecophysiological stress responses at higher biological levels. This reveals considerable promise for using acute molecular responses as a guide to possible chronic impact on populations of environmental stress. Ultimately, this could improve current environmental risk assessment through providing early 'signposts' (*sensu *Hutchinson and coworkers 2006 [48]) to the need for higher tier testing or other appropriate actions.

## Materials and methods

### Microarray experiment

*D. magna *were obtained from the Water Research Centre (Medmenham, UK) and were cultured at the University of Reading for more than 2 years before the experiment. Full details of culturing methods were reported by Hooper and coworkers [49]. Tests were conducted in 5 l glass aquaria (height 22 cm, internal diameter 18.5 cm, and thickness 5 mm; Harzkristall GmbH, Derenburg, Germany) at 20 ± 1°C and a 16:8 light:dark photoperiod. During the first 24 hours the aquaria contained an inner exposure vessel (height 13 cm and diameter 9 cm) with a nylon mesh bottom to allow free movement of the test media between the two vessels. Quadruplicates were assigned in a randomized block design and initiated with 310 fourth brood neonates (<24 hours old) that were exposed to a control or one of three ibuprofen concentrations (20, 40, and 80 mg/l ibuprofen), applied as ibuprofen-sodium (Sigma-Aldrich, Gillingham, UK; CAS number 31121-93-4; batch number 64K0892) in reconstituted water. Following 24 hours of exposure, ten individuals were transferred to the outer aquarium for a chronic population study, described in detail by Heckmann and coworkers [11], while the inner vessel with the remaining 300 neonates was removed. These neonates were stored in RNA *later*^® ^(Ambion, Warrington, UK) at -80°C for subsequent RNA extractions. A reference pool of approximately 6,000 *D. magna *that were under 48 hours old was obtained from the same brood as those exposed. Ibuprofen was sampled (1.5 ml) for quantification from every replicate of each treatment at time zero and at 24 hours. Subsequent analysis, using UV spectrophotometry [21], revealed that the difference between nominal and measured concentrations was under 10%, except for one replicate of 20 mg/l ibuprofen, which was under 20% at 24 hours. Further details on water chemistry (conductivity, dissolved oxygen, and pH) are available in the report by Heckmann and coworkers [11].

### Microarray hybridization

Hybridization followed a reference pool design in which each experimental sample was hybridized against a common reference pool sample. Total RNA was extracted using the RNeasy Mini kit with on-column DNase treatment (Qiagen, Crawley, UK) in order to remove any traces of genomic DNA, following the manufacturer's instructions. RNA concentrations were determined by spectrophotometry using GeneQuant Pro (Biochrom, Cambridge, UK), and RNA integrity was verified using a BioAnalyzer 2100 (Agilent Technologies, Stockport, UK). cDNA was synthesized from 17.5 μg total RNA (treatment and reference pool material, respectively) and labeled with Alexa Fluor^® ^dyes (two-colour reference design: Alexa Fluor^® ^647 and Alexa Fluor^® ^555 for experimental and reference pool samples, respectively) using SuperScript™ Plus Indirect cDNA Labeling System (Invitrogen, Paisley, UK). Slides were pre-hybridized in a solution containing 50% vol/vol de-ionized formamide, 5× sodium chloride-sodium citrate, 0.1% sodium dodecyl sulfate and 1% weight/vol bovine serum albumin (Sigma-Aldrich, Warrington, UK), and incubated at 42°C in a Techne HB-1 Hybridiser (Techne Ltd, Stone, UK) for 1 h.

A 45 μl hybridization probe solution was prepared with 22.5 μl de-ionized formamide, 5× sodium chloride-sodium citrate, the labeled cDNA mix (combined experimental sample and reference pool cDNA), and a hybridization block mix containing 0.1% sodium dodecyl sulfate, 0.5 mg/ml polyA RNA (Sigma-Aldrich, Warrington, UK), 0.5 mg/ml yeast tRNA, 0.5 mg/ml salmon sperm DNA, and 25 μg/ml human and 25 μg/ml mouse *Cot-1 *DNA (Invitrogen Paisley, UK). The probes were hybridized to individual microarray slides (one hybridization was performed for each slide; *n *= 16) under a 2560 lifterslip™ (Implen, Southend on Sea, UK). The slides were hybridized in batches of four slides corresponding to the control and respective ibuprofen treatments within a biological replicate. The slides were then placed in an airtight plastic box and incubated at 42°C in a Techne HB-1 Hybridiser (Techne Ltd, Stone, UK) for 16 hours. Details of pre-hybridization and post-hybridization washes, and construction of the microarray are described in Additional data file 5.

### Microarray analysis

Microarray slides were scanned using a GenePix 4200A microarray scanner (Axon Instruments, Inverurie, UK) installed with GenePix^® ^Pro 5.0. The data were normalized per slide to the median of ratios using spots with a regression ratio above 0.7, a sum of medians above 500, a saturation value below 3, and a signal to noise ratio of 3 or greater [50]. Overall, some 15% of the spots per chip were flagged as 'present' based on these criteria, and they were utilized to calculate normalization factors [50]. Regrettably, one slide failed (80 mg/l ibuprofen; replicate 2) and was omitted from further analysis. Only spots flagged as present and/or marginal in 80% of the arrays were analyzed (7,135 spots).

Analysis followed the protocol of Connon and coworkers [19]. Tab-delimited text files from GenePix were imported to GeneSpring 7.2 (Agilent Technologies, Santa Clara CA, USA). Median signal and control channels (F635 and F532) were used to calculate working ratios in GeneSpring. Data were normalized using per spot and per chip intensity-dependent (Global LOWESS) normalization, followed by a per gene normalization to the control samples, within each hybridization batch, to normalize for batch variations (the ibuprofen treatments within each biological replicate were normalised to the control sample of the same biological replicate; *n *= 4). MA plots of raw and normalized data are available in Additional data file 6, which shows data quality before and after normalization. Following data normalization, spots with expression levels between 0.714 and 1.4 in all conditions (4,912 spots) were removed from further analysis using GeneSpring filters (resulting in 2,223 spots) [51]. Two sample independent *t*-tests (equal variances assumed) were carried out on log_2 _ratios between control and ibuprofen treatments. This filtering step ensured that only spots that changed in at least one concentration were subjected to further analysis. The resulting *t*-test gene lists were then merged (827 spots) and subjected to a one-way analysis of variance (equal variances not assumed) with no multiple testing corrections, resulting in a list of 272 spots. For all statistical tests, a significance level of 5% was applied.

### Annotation

Basic local alignment search tool (BLAST) analyses were conducted between August 2006 and March 2007 on fragments that responded significantly to the exposure treatment. Sequences were annotated according to BLASTX homology search against GenBank [52], UniProt [53], and InterPro [54]. Sequences were only annotated if they had a BLAST hit with an expect value (E value) below 10^-5 ^and a score above 50. GeneBank/UniProt accession number and species' match were recorded with each annotation (see Additional data file 1).

### MIAME (minimum information about a microarray experiment) compliance

Available *D. magna *sequences can be found at DaphniaBase [55] and from the website of the *Daphnia *research group of the University of Reading [56]. Microarray images and data are accessible through the public repository Array Express at the European Bioinformatics Institute (accession number: E-MAXD-20). Microarray images and normalized expression data were also catalogued on our website [56].

### Follow-up experiment assessing chronic phenotypic responses

The experiment was based on a randomized block design with five replicates of a control and three treatments with ibuprofen-sodium (Sigma-Aldrich, Warrington, UK: CAS number 31121-93-4; batch number 64K0892) containing 20, 40 and 80 mg/l ibuprofen, respectively. Each replicate consisted of one adult (14 days old) placed in a 1,000 ml glass beaker containing 1 l reconstituted freshwater (see Hooper and coworkers [49]), with or without the addition of ibuprofen. The test vessels were kept in a 20 ± 1°C temperature-controlled room with a light:dark regimen of 16:8 hours. Adults were exposed to ibuprofen for 8 days and were fed daily with equal amounts of green algae *Chlorella vulgaris *var *viridis *(equivalent to 1.00 mg/day carbon). Measured biological end-points are displayed in Table 2. To assess the potential induction of male offspring caused by maternal exposure to ibuprofen, a total of 20 fourth and fifth brood offspring from each treatment were transferred to 2 l plastic beakers with 1.2 l of uncontaminated culture media, except in the 80 mg/l ibuprofen treatment were zero, and five offspring were produced in each of the fourth and fifth broods. Fourth and fifth brood neonates were reared like normal cultures (see Hooper and coworkers [49]) until they reached sexual maturity (approximately 9 days). No males were present in either of the treatments. However, 60% mortality was observed among fifth brood neonates that had been maternally exposed to 80 mg/l ibuprofen. There was no mortality among the other neonates or during the exposure of adults.

Ibuprofen was sampled (1.5 ml) from each replicate on days 0 and 8 (adult exposure only), and subsequent quantifications revealed that the difference between nominal and measured concentrations was under 10%. Water temperature was checked daily and averaged 19.6 ± 0.2°C (mean ± standard error; *n *= 60) throughout the experimental period. Other measured water chemistry parameters are available in Additional data file 7. Corresponding with our previous studies [11], both pH and conductivity were slightly but significantly (*P *< 0.05, analysis of variance) increased with increasing ibuprofen concentration.

### Follow-up experiment assessing temporal expression of key genes

The experiment comprised a control and one concentration of ibuprofen-sodium (Sigma-Aldrich, Warrington, UK: CAS number 31121-93-4; batch number 64K0892) containing 80 mg/l ibuprofen. Fifty third-brood neonates (<24 hours old) were placed in 150 ml glass beakers containing 100 ml reconstituted freshwater with or without the addition of ibuprofen. Test conditions were as above but without feeding. The neonates were exposed to ibuprofen for 2, 4, 8, 24, or 48 hours. Each treatment and time point were replicated four times and assigned to a randomized block design. Ibuprofen was sampled (1.5 ml) from each replicate at every time point, and the difference between nominal and measured concentrations was under 10%. Water temperature averaged 19.9 ± 1.1°C (mean ± standard error; *n *= 8) throughout the experimental period. Other water chemistry parameters were measured at every time point from pooled samples of the same treatment (see Additional data file 7).

Following exposure (2, 4, 8, 24, and 48 hours), neonates were immediately transferred to 0.2 ml RNA *later*^® ^(Ambion, Warrington, UK) using our recently developed methodology [57]. Samples were stored at -80°C and total RNA was subsequently extracted and processed as previously described [21]. cDNA was synthesized from 1 μg total RNA and diluted 10-fold, resulting in total RNA concentrations of 5 ng/μl, and stored at -20°C. Primers were designed using Primer3 [58] and synthesized by MWG (Ebersberg, Germany; see Additional data file 8). QPCR was conducted on the GeneAmp 5700 Sequence Detection System (Applied Biosystems) using ABsolute™ QPCR SYBR^® ^Green ROX (500 nmol/l) mix (ABgene, Epsom, UK). Each reaction was run in duplicate and contained 2.5 μl cDNA template (equivalent to 12.5 ng total RNA) along with 900 nmol/l primers in a final volume of 25 μl. Cycling parameters were 95°C for 15 minutes to activate the DNA polymerase, then 40 cycles of 95°C for 15 seconds and 60°C for 1 minute. Melting curves were performed by using dissociation curve Sequence Detection System software version 1.3 (Applied Biosystems) to verify that only a single product with no primer-dimers was amplified. QPCR data processing and statistical analysis were performed as previously reported [21] using DART-PCR [59] and geNorm [60].

## Abbreviations

AA, arachidonic acid; BLAST, basic local alignment search tool; COX, cycloxygenase; GO, Gene Ontology; JH, juvenile hormone; JHA, juvenile hormone analog; KEGG, Kyoto Encyclopedia of Genes and Genomes; LOX, lipoxygenase; LT, leukotriene; LTB_4_DH, leukotriene B_4 _12-hydroxydehydrogenase; MOA, mode of action; NSAID, nonsteroidal anti-inflammatory drug; PCR, polymerase chain reaction; PG, prostaglandin; PPAR, peroxisome proliferator-activated receptor; QPCR, quantitative PCR.

## Authors' contributions

All authors were involved in designing the experiments. RC, HLH, CJH, and AB assisted LHH in conducting parts of the practical experimental work. LHH performed the statistical analyses and drafted the main manuscript under the supervision of AC, RMS, THH, and SJM. Methods on microarray hybridization, and analysis and supplementary methods on microarray construction were drafted by LHH and RC. All authors read, contributed intellectually toward, and approved the final manuscript.

## Additional data files

The following additional data are available with the online version of this paper. Additional data file 1 lists all of the cDNAs (annotated) that responded to ibuprofen treatment on the *D. magna *microarray. Additional data file 2 shows the relative expression and GO of the unique *D. magna *genes responding to acute ibuprofen exposure. Additional data file 3 displays QPCR confirmation of selected *D. magna *genes responding on the cDNA microarray. Additional data file 4 shows an image of a *D. magna *embryo arrested at developmental stage 1 to 2 after maternal exposure to ibuprofen. Additional data file 5 provides supplementary methods on microarray hybridization and microarray construction. Additional data file 6 shows MA plots of raw and normalized microarray data. Additional data file 7 shows water chemical parameters measured during the follow-up experiments. Additional data file 8 lists technical data on QPCR (for example, primers and amplification efficiency) from the follow-up experiment assessing temporal expression of key genes responding to ibuprofen.

## Supplementary Material

Additional data file 1 Presented is a table listing all the cDNAs (annotated) that responded to ibuprofen treatment on the *D. magna *microarray. Click here for file

Additional data file 2 Presented is a table showing the relative expression and GO of the unique *D. magna *genes responding to acute ibuprofen exposure. Click here for file

Additional data file 3 Presented is a table displaying QPCR confirmation of selected *D. magna *genes responding on the cDNA microarray. Click here for file

Additional data file 4 Presented is an image of a *D. magna *embryo arrested at developmental stage 1 to 2 after maternal exposure to ibuprofen. Click here for file

Additional data file 5 Presented is a document with supplementary methods on microarray hybridization and microarray construction. Click here for file

Additional data file 6 Presented are MA plots of raw and normalized microarray data Click here for file

Additional data file 7 Presented is a table showing water chemical parameters measured during the follow-up experiments. Click here for file

Additional data file 8 Presented is a table listing technical data on QPCR (for instance, primers and amplification efficiency) from the follow-up experiment assessing temporal expression of key genes responding to ibuprofen. Click here for file

## References

[B1] Walker CH, Hopkin SP, Sibly RM, Peakall DB (2006). Principles of Ecotoxicology.

[B2] Chen DR, Toone WM, Mata J, Lyne R, Burns G, Kivinen K, Brazma A, Jones N, Bahler J (2003). Global transcriptional responses of fission yeast to environmental stress.. Mol Biol Cell.

[B3] Gasch AP, Spellman PT, Kao CM, Carmel-Harel O, Eisen MB, Storz G, Botstein D, Brown PO (2000). Genomic expression programs in the response of yeast cells to environmental changes.. Mol Biol Cell.

[B4] Ashburner M, Ball CA, Blake JA, Botstein D, Butler H, Cherry JM, Davis AP, Dolinski K, Dwight SS, Eppig JT, Harris MA, Hill DP, Issel-Tarver L, Kasarskis A, Lewis S, Matese JC, Richardson JE, Ringwald M, Rubin GM, Sherlock G (2000). Gene Ontology: tool for the unification of biology.. Nat Genet.

[B5] Kanehisa M, Goto S (2000). KEGG: Kyoto Encyclopedia of Genes and Genomes.. Nucleic Acids Res.

[B6] Poynton HC, Varshavsky JR, Chang B, Cavigiolio G, Chan S, Holman PS, Loguinov AV, Bauer DJ, Komachi K, Theil EC, Perkins EJ, Hughes O, Vulpe CD (2007). *Daphnia magna *ecotoxicogenomics provides mechanistic insights into metal toxicity.. Environ Sci Technol.

[B7] Soetaert A, Moens LN, van der Ven K, van Leemput K, Naudts B, Blust R, De Coen WM (2006). Molecular impact of propiconazole on *Daphnia magna *using a reproduction-related cDNA array.. Comp Biochem Physiol C Toxicol Pharmacol.

[B8] Watanabe H, Takahashi E, Nakamura Y, Oda S, Tatarazako N, Iguchi T (2007). Development of a *Daphnia magna *DNA microarray for evaluating the toxicity of environmental chemicals.. Environ Toxicol Chem.

[B9] Terashima J, Bownes M (2005). A microarray analysis of genes involved in relating egg production to nutritional intake in *Drosophila melanogaster*.. Cell Death Differ.

[B10] Korsloot A, van Gestel CAM, van Straalen NM (2004). Environmental Stress and Cellular Response in Arthropods.

[B11] Heckmann L-H, Callaghan A, Hooper HL, Connon R, Hutchinson TH, Maund SJ, Sibly RM (2007). Chronic toxicity of ibuprofen to *Daphnia magna*: effects on life history traits and population dynamics.. Toxicol Lett.

[B12] Stanley DW (2000). Eicosanoids in Invertebrate Signal Transduction Systems.

[B13] JGI Genome Portal. http://www.jgi.doe.gov/Daphnia.

[B14] Shaw JR, Pfrender ME, Eads BD, Klaper R, Callaghan A, Colson I, Gilbert DG, Colbourne JK, Kille P, Hogstrand C *Daphnia *as an emerging model for toxicological genomics.. Advances in Experimental Biology in Toxicogenomics.

[B15] Oleksiak MF, Roach JL, Crawford DL (2005). Natural variation in cardiac metabolism and gene expression in *Fundulus heteroclitus*.. Nat Genet.

[B16] Feder ME, Mitchell-Olds T (2003). Evolutionary and ecological functional genomics.. Nat Rev Genet.

[B17] Hutchinson TH (2007). Small is useful in endocrine disrupter assessment - four key recommendations for aquatic invertebrate research.. Ecotoxicology.

[B18] Watanabe H, Tatarazako N, Oda S, Nishide H, Uchiyama I, Morita M, Iguchi T (2005). Analysis of expressed sequence tags of the water flea *Daphnia magna*.. Genome.

[B19] Connon R, Hooper HL, Sibly RM, Lim FL, Heckmann L-H, Moore DJ, Watanabe H, Soetaert A, Cook K, Maund SJ, Hutchinson TH, Moggs J, De Coen W, Iguchi T, Callaghan A (2008). Linking molecular and population stress responses of *Daphnia magna *exposed to cadmium.. Environ Sci Technol.

[B20] Fujimoto Y, Shimada S, Fujikawa T, Sakuma S, Fujita T (1991). Triacylglycerol lipase mediated release of arachidonic acid for prostaglandin synthesis in rabbit kidney medulla microsomes.. Prostaglandins Leukot Essent Fatty Acids.

[B21] Heckmann L-H, Connon R, Hutchinson TH, Maund SJ, Sibly RM, Callaghan A (2006). Expression of target and reference genes in *Daphnia magna *exposed to ibuprofen.. BMC Genomics.

[B22] Tokishita S, Kato Y, Kobayashi T, Nakamura S, Ohta T, Yamagata H (2006). Organization and repression by juvenile hormone of a vitellogenin gene cluster in the crustacean, *Daphnia magna*.. Biochem Biophys Res Commun.

[B23] Gilbert LI, Granger NA, Roe RM (2000). The juvenile hormones: historical facts and speculations on future research directions.. Insect Biochem Mol Biol.

[B24] Rider CV, Gorr TA, Olmstead AW, Wasilak BA, Leblanc GA (2005). Stress signaling: coregulation of hemoglobin and male sex determination through a terpenoid signaling pathway in a crustacean.. J Exp Biol.

[B25] Gorr TA, Rider CV, Wang HY, Olmstead AW, LeBlanc GA (2006). A candidate juvenoid hormone receptor cis-element in the *Daphnia magna *hb2 hemoglobin gene promoter.. Mol Cell Endocrinol.

[B26] Wang YH, Wang GR, LeBlanc GA (2007). Cloning and characterization of the retinoid × receptor from a primitive crustacean *Daphnia magna*.. Gen Comp Endocrinol.

[B27] Merzendorfer H, Zimoch L (2003). Chitin metabolism in insects: structure, function and regulation of chitin synthases and chitinases.. J Exp Biol.

[B28] Sricharoen S, Kim JJ, Tunkijjanukij S, Soderhall I (2005). Exocytosis and proteomic analysis of the vesicle content of granular hemocytes from a crayfish.. Dev Comp Immunol.

[B29] Hayashi Y, Heckmann L-H, Callaghan A, Sibly RM (2008). Reproduction recovery of the crustacean *Daphnia magna *after chronic exposure to ibuprofen.. Ecotoxicology.

[B30] Kast-Hutcheson K, Rider CV, LeBlanc GA (2001). The fungicide propiconazole interferes with embryonic development of the crustacean *Daphnia magna*.. Environ Toxicol Chem.

[B31] Heckmann L-H, Maraldo K, Krogh PH (2005). Life stage specific impact of dimethoate on the predatory mite *Hypoaspis aculeifer *Canestrini (Gamasida: Laelapidae).. Environ Sci Technol.

[B32] English N, Hughes V, Wolf GR (1996). Induction of cytochrome P-450(BM-3) (CYP 102) by non-steroidal anti-inflammatory drugs in *Bacillus megaterium*.. Biochem J.

[B33] Erriquez J, Gilardino A, Ariano P, Munaron L, Lovisolo D, Distasi C (2005). Calcium signals activated by arachidonic acid in embryonic chick ciliary ganglion neurons.. Neurosignals.

[B34] Funk CD (2001). Prostaglandins and leukotrienes: advances in eicosanoid biology.. Science.

[B35] Feige JN, Gelman L, Michalik L, Desvergne B, Wahli W (2006). From molecular action to physiological outputs: peroxisome proliferator-activated receptors are nuclear receptors at the crossroads of key cellular functions.. Prog Lipid Res.

[B36] Mark M, Ghyselinck NB, Chambon P (2006). Function of retinoid nuclear receptors: lessons from genetic and pharmacological dissections of the retinoic acid signaling pathway during mouse embryogenesis.. Annu Rev Pharmacol Toxicol.

[B37] Hori T, Yokomizo T, Ago H, Sugahara M, Ueno G, Yamamoto M, Kumasaka T, Shimizu T, Miyano M (2004). Structural basis of leukotriene B-4 12-hydroxydehydrogenase/15-oxo-prostaglandin 13-reductase catalytic mechanism and a possible Src homology 3 domain binding loop.. J Biol Chem.

[B38] Clish CB, Sun Y-P, Serhan CN (2001). Identification of dual cyclooxygenase-eicosanoid oxidoreductase inhibitors: NSAIDs that inhibit PG-LX reductase/LTB4 dehydrogenase.. Biochem Biophys Res Commun.

[B39] Medeiros MN, Mendonca LH, Hunter AL, Paiva-Silva GO, Mello FG, Henze IP, Masuda H, Maya-Monteiro CM, Machado EA (2004). The role of lipoxygenase products on the endocytosis of yolk proteins in insects: participation of cAMP.. Arch Insect Biochem Physiol.

[B40] Silver RB, Oblak JB, Jeun GS, Sung JJ, Dutta TC (1994). Leukotriene B4, an arachidonic acid metabolite, regulates intracellular free calcium release in eggs and mitotic cells of the sand dollar (*Echinaracnius parma*).. Biol Bull.

[B41] Tatarazako N, Oda S, Watanabe H, Morita M, Iguchi T (2003). Juvenile hormone agonists affect the occurrence of male *Daphnia*.. Chemosphere.

[B42] LeBlanc GA (2007). Crustacean endocrine toxicology: a review.. Ecotoxicology.

[B43] Ye XQ, Hama K, Contos JJA, Anliker B, Inoue A, Skinner MK, Suzuki H, Amano T, Kennedy G, Arai H, Aoki J, Chun J (2005). LPA(3)-mediated lysophosphatidic acid signalling in embryo implantation and spacing.. Nature.

[B44] Spaziani EP, Hinsch GW, Edwards SC (1993). Changes in prostaglandin E_2 _and F2-alpha during vitellogenesis in the Florida crayfish *Procambarus paeninsulanus*.. J Comp Physiol B.

[B45] Sagi A, Silkovsky J, Fleisher-Berkovich S, Danon A, Chayoth R (1995). Prostaglandin E_2 _in previtellogenic ovaries of the prawn *Macrobrachium rosenbergii*: synthesis and effect on the level of cAMP.. Gen Comp Endocrinol.

[B46] Corona M, Velarde RA, Remolina S, Moran-Lauter A, Wang Y, Hughes KA, Robinson GE (2007). Vitellogenin, juvenile hormone, insulin signaling, and queen honey bee longevity.. Proc Natl Acad Sci USA.

[B47] Kimura KD, Tissenbaum HA, Liu YX, Ruvkun G (1997). daf-2, an insulin receptor-like gene that regulates longevity and diapause in *Caenorhabditis elegans*.. Science.

[B48] Hutchinson TH, Ankley GT, Segner H, Tyler CR (2006). Screening and testing for endocrine disruption in fish: biomarkers as 'signposts', not 'traffic lights', in risk assessment.. Environ Health Perspect.

[B49] Hooper HL, Connon R, Callaghan A, Maund SJ, Liess M, Duquesne S, Hutchinson TH, Moggs JG, Sibly RM (2006). The use of image analysis methods to estimate population growth rate in *Daphnia magna*.. J Appl Ecol.

[B50] Verdnik D (2004). Guide to Microarray Enalysis.

[B51] GeneSpring. http://www.chem.agilent.com/scripts/pds.asp?lpage=27881.

[B52] GenBank. http://www.ncbi.nlm.nih.gov/Genbank/.

[B53] UniProt. http://www.ebi.ac.uk/uniprot.

[B54] InterPro. http://www.ebi.ac.uk/interpro/.

[B55] DaphniaBase. http://daphnia.nibb.ac.jp.

[B56] *Daphnia *research group of the University of Reading. http://www.biosci.rdg.ac.uk/Research/eb/daphnia.htm.

[B57] Heckmann L-H, Bouetard A, Hill CJ, Sibly RM, Callaghan A (2007). A simple and rapid method for preserving RNA of aquatic invertebrates for ecotoxicogenomics.. Ecotoxicology.

[B58] Primer3. http://frodo.wi.mit.edu/cgi-bin/primer3/primer3_www.cgi.

[B59] Peirson SN, Butler JN, Foster RG (2003). Experimental validation of novel and conventional approaches to quantitative real-time PCR data analysis.. Nucleic Acids Research.

[B60] Vandesompele J, De Preter K, Pattyn F, Poppe B, Van Roy N, De Paepe A, Speleman F (2002). Accurate normalization of real-time quantitative RT-PCR data by geometric averaging of multiple internal control genes.. Genome Biol.

