# Supplementary material for: Systems biology meets stress ecology: linking molecular and organismal stress responses in Daphnia magna
Source: Genome Biol. 2008 Feb 21;9(2):R40. doi: 10.1186/gb-2008-9-2-r40 (PMC2374704; doi:10.1186/gb-2008-9-2-r40)
Supplement: Additional data file 1 — Presented is a table listing all the cDNAs (annotated) that responded to ibuprofen treatment on the D. magna microarray. [file gb-2008-9-2-r40-S1.pdf]

Additional data file 1. Annotation of microarray *Daphnia magna* genes responding to acute ibuprofen exposure.

Clone ID[a]	Putative gene	Gene symbol	Species Match[b]	Accession#	E-value	Contig[c]
IGU001_0032_G10	*16S ribosomal RNA*	*16S rRNA*	*Daphnia magna*	AY921452	0.00E+00	1130
IGU001_0035_F02	*16S ribosomal RNA*	*16S rRNA*	*Daphnia magna*	AY921452	0.00E+00	1130
IGU001_0049_B12	*16S ribosomal RNA*	*16S rRNA*	*Daphnia magna*	AY921452	0.00E+00	1130
IGU001_0053_A01	*16S ribosomal RNA*	*16S rRNA*	*Daphnia magna*	AY921452	0.00E+00	1130
R07CDR3E07	*16S ribosomal RNA*	*16S rRNA*	*Daphnia magna*	AY921452	0.00E+00	1130
WTH001_0003_C20	*16S ribosomal RNA*	*16S rRNA*	*Daphnia magna*	AY921452	0.00E+00	1130
WTH001_0003_L15	*16S ribosomal RNA*	*16S rRNA*	*Daphnia magna*	AY921452	0.00E+00	1130
WTH001_0012_A16	*16S ribosomal RNA*	*16S rRNA*	*Daphnia magna*	AY921452	0.00E+00	1130
WTH001_0002_J23	*28S ribosomal RNA*	*28S rRNA*	*Daphnia magna*	AF346515	0.00E+00	128
WTH001_0009_H09	*28S ribosomal RNA*	*28S rRNA*	*Daphnia magna*	AF346515	0.00E+00	128
WTH001_0014_N15	*28S ribosomal RNA*	*28S rRNA*	*Daphnia magna*	AF346515	2.00E-70	128
IGU001_0051_A10	*5'-nucleotidase-related*	*Nt5*	*Anopheles gambiae*	XP_308620	1.00E-24	
R14PHR1A09	*Actin*	*Act*	*Daphnia magna*	CAB99474	1.00E-70	
WTH001_0007_L13	*Actin*	*Act*	*Apis mellifera*	XP_001121105	7.00E-23	3
WTH001_0009_A05	*Acyl CoA synthetase*	*ACS*	*Apis mellifera*	XP_395996	8.00E-34	671
IGU001_0034_C11	*ADP/ATP translocase*	*ANT*	*Aedes aegypti*	ABF18037	3.00E-139	49
WTH001_0009_O02	*Alpha amylase*	*amyA*	*Corbicula fluminea*	AAO17927	3.00E-36	
WTH001_0005_C13	*Astacin (zinc metalloprotease)*	*ASTFL*	*Drosophila melanogaster*	AAM29490	2.00E-33	809
IGU001_0002_E04	*ATP synthase a chain*	*atpB*	*Daphnia pulex*	NP_008625	4.00E-07	
IGU001_0048_A07	*ATP synthase a chain*	*atpB*	*Daphnia melanica*	ABD19465	9.00E-28	
WTH001_0011_G12	*ATP synthase a chain*	*atpB*	*Daphnia melanica*	ABD19465	1.00E-22	
WTH001_0009_J10	*Bovine pancreatic trypsin inhibitor domain*	*BPTI*	***Danio rerio***	AAI15336	2.00E-18	
IGU001_0028_A09	*Calcification-associated peptide-2 precursor*	*cap-2*	*Tribolium castaneum*	XP_973761	8.00E-08	106
WTH001_0012_E06	*Calcification-associated peptide-2 precursor*	*cap-2*	*Tribolium castaneum*	XP_973761	8.00E-08	106
WTH001_0002_H09	*Carboxypeptidase A2*	*CPA2*	*Strongylocentrotus purpuratus*	XP_786036	3.00E-21	800
WTH001_0007_J09	*Cathepsin L-like protease precursor*	*CATL1*	*Artemia franciscana*	AAV63977	3.00E-47	389
WTH001_0011_J11	*Cathepsin L-like protease precursor*	*CATL1*	*Artemia franciscana*	AAD39513	7.00E-24	389
WTH001_0003_J14	*Chitinase*	*Cht*	*Tribolium castaneum*	ABG47448	7.00E-73	961
WTH001_0006_H05	*Chitinase*	*Cht*	*Apis mellifera*	XP_395734	3.00E-22	961
WTH001_0007_H23	*Chitinase*	*Cht*	*Apis mellifera*	XP_395734	2.00E-33	961
WTH001_0007_N19	*Chitinase*	*Cht*	*Litopenaeus vannamei*	CAA75309	1.00E-41	961
WTH001_0009_A13	*Chitinase*	*Cht*	*Dermatophagoides pteronyssinus*	AAY84564	7.00E-34	961
WTH001_0009_G16	*Chitinase*	*Cht*	*Anopheles gambiae*	EAT45796	3.00E-32	961
WTH001_0009_M05	*Chitinase*	*Cht*	*Drosophila melanogaster*	AAM50881	3.00E-26	961
WTH001_0009_N10	*Chitinase*	*Cht*	*Apis mellifera*	XP_395734	2.00E-38	961
WTH001_0010_O15	*Chitinase*	*Cht*	*Ixodes ricinus*	AAQ93649	2.00E-18	961
IGU001_0028_B02	*Chitinase*	*Cht*	*Tenebrio molitor*	AAP92418	2.00E-36	132

WTH001_0003_F07	*Chitin-binding domain type 2*	*ChtBD2*	*Aedes aegypti*	EAT45952	2.00E-88	109
WTH001_0002_G19	*Chitin-binding Peritrophin-A domain*	*Peritrophin-A*	*Apis mellifera*	XP_001120217	8.00E-35	957
IGU001_0017_B10	*Chymotrypsin B2*	*Ctrb2*	*Litopenaeus vannamei*	CAA71673	1.00E-36	384
IGU001_0044_G03	*Chymotrypsin B2*	*Ctrb2*	*Litopenaeus vannamei*	CAA71673	9.00E-33	384
WTH001_0005_A17	*Contactin 1*	*Cntn1*	***Macaca mulatta***	XP_001088804	7.00E-06	
IGU001_0028_H04	*C-terminal binding protein*	*CTBP*	*Tribolium castaneum*	XP_972241	1.00E-74	
IGU001_0026_E10	*C-type lectin-like*	*CLECT*	*Incilaria fruhstorferi*	BAA19861	1.00E-05	960
IGU001_0017_H07	*CUB domain*	*CUB*	*Tribolium castaneum*	XP_973589	3.00E-20	1344
WTH001_0002_N09	*Cuticle protein 7*	*CP7*	*Blaberus craniifer*	P82120	1.00E-18	771
WTH001_0014_K24	*Cuticle protein 7*	*CP7*	*Blaberus craniifer*	P82120	1.00E-18	771
WTH001_0009_O15	*Cuticle structural protein post-ecdysial PCP16.7*	*PCP16.7*	*Tenebrio molitor*	S78003	2.00E-15	1
IGU001_0030_H07	*Cuticular protein precursor*	*LPCP29*	*Anopheles gambiae*	EAA14439	2.00E-11	561
WTH001_0002_A20	*Cuticular protein precursor*	*LPCP29*	*Tenebrio molitor*	CAA03880	4.00E-10	561
WTH001_0013_K04	*Cysteine-rich intestinal protein*	*CRIP*	*Apis mellifera*	XP_001122454	1.00E-32	652
IGU001_0002_C09	*Cytochrome b*	*CYTB*	*Daphnia pulex*	ABD19355	2.00E-60	
IGU001_0029_H01	*Cytochrome b*	*CYTB*	*Daphnia pulex*	NP_008632	6.00E-58	182
IGU001_0031_D07	*Cytochrome b*	*CYTB*	*Daphnia pulex*	NP_008632	9.00E-72	182
IGU001_0007_G01	*Cytochrome c oxidase subunit 1*	*CO1*	*Daphnia pulex*	NP_008622	1.00E-40	135
IGU001_0007_G12	*Cytochrome c oxidase subunit 1*	*CO1*	*Daphnia pulex*	ABD19215	1.00E-60	135
IGU001_0015_H03	*Cytochrome c oxidase subunit 1*	*CO1*	*Daphnia pulex*	NP_008622	4.00E-61	135
IGU001_0034_H02	*Cytochrome c oxidase subunit 1*	*CO1*	*Daphnia pulex*	ABD19215	2.00E-55	135
IGU001_0050_H02	*Cytochrome c oxidase subunit 1*	*CO1*	*Daphnia pulex*	AAB53197	2.00E-40	135
WTH001_0003_L17	*Cytochrome c oxidase subunit 1*	*CO1*	*Daphnia pulex*	NP_008622	3.00E-37	135
IGU001_0012_A03	*Cytochrome c oxidase subunit 2*	*CO2*	*Daphnia pulex*	ABD19294	1.00E-51	1020
IGU001_0014_B12	*Cytochrome c oxidase subunit 2*	*CO2*	*Daphnia pulex*	ABD19294	4.00E-51	
IGU001_0027_E12	*Cytochrome c oxidase subunit 2*	*CO2*	*Daphnia pulex*	ABD19294	6.00E-61	1020
WTH001_0007_G08	*Cytochrome c oxidase subunit 2*	*CO2*	*Daphnia pulex*	NP_008623	1.00E-21	1020
WTH001_0010_G24	*DD5 (structural constituent of cuticle)*	*DD5*	*Marsupenaeus japonicus*	AB049147	1.00E-06	327
WTH001_0001_K09	*DEAD Box ATP-dependent RNA helicase*	*DEADc*	*Apis mellifera*	XP_394723	2.00E-45	968
WTH001_0002_F01	*Endo-beta-1,4-mannanase*	*MANA*	*Mytilus edulis*	AJ271365	3.00E-22	
IGU001_0032_A08	*Enolase*	*Eno*	*Daphnia magna*	AAS02302	0.00E+00	511
WTH001_0001_D10	*Enolase*	*Eno*	*Daphnia magna*	AY522935	0.00E+00	511
WTH001_0001_N23	*Enolase*	*Eno*	*Daphnia magna*	AAS02302	0.00E+00	511
R14PHF2B06	*Fatty acid binding protein 3*	*FABP3*	*Schistocerca gregaria*	AAK20174	9.00E-11	
IGU001_0025_E01	*Ferritin*	*Fer*	*Daphnia pulex*	CAB72315	9.00E-51	329
R09CAF1A02	*Ferritin*	*Fer*	*Daphnia pulex*	CAB72315	4.00E-64	
R09CAF1E02	*Ferritin*	*Fer*	*Daphnia pulex*	CAB72315	2.00E-63	
RBH01	*Ferritin*	*Fer*	*Daphnia pulex*	CAB72315	8.00E-49	
WTH001_0001_K10	*Ferritin*	*Fer*	*Daphnia pulex*	AJ245734	2.00E-17	329
WTH001_0001_N01	*Ferritin*	*Fer*	*Daphnia pulex*	AJ245734	7.00E-45	329

WTH001_0002_E17	*Ferritin*	*Fer*	*Daphnia pulex*	ABK91576	2.00E-44	329
WTH001_0002_E23	*Ferritin*	*Fer*	*Daphnia pulex*	ABK91576	2.00E-72	329
WTH001_0002_O01	*Ferritin*	*Fer*	*Daphnia pulex*	ABK91576	2.00E-72	329
WTH001_0003_M01	*Ferritin*	*Fer*	*Daphnia pulex*	ABK91576	2.00E-72	329
WTH001_0004_K17	*Ferritin*	*Fer*	*Daphnia pulex*	ABK91576	2.00E-72	329
WTH001_0004_M03	*Ferritin*	*Fer*	*Daphnia pulex*	CAB72315	1.00E-49	
WTH001_0005_O20	*Ferritin*	*Fer*	*Daphnia pulex*	ABK91576	2.00E-72	329
WTH001_0009_A17	*Ferritin*	*Fer*	*Daphnia pulex*	ABK91576	2.00E-72	329
WTH001_0009_K02	*Ferritin*	*Fer*	*Daphnia pulex*	ABK91576	2.00E-72	329
WTH001_0010_B18	*Ferritin*	*Fer*	*Daphnia pulex*	ABK91576	7.00E-10	329
WTH001_0010_E20	*Ferritin*	*Fer*	*Daphnia pulex*	ABK91576	5.00E-59	329
WTH001_0010_G18	*Ferritin*	*Fer*	*Daphnia pulex*	ABK91576	2.00E-72	329
WTH001_0010_L19	*Ferritin*	*Fer*	*Daphnia pulex*	ABK91576	2.00E-72	329
WTH001_0010_N23	*Ferritin*	*Fer*	*Daphnia pulex*	ABK91576	2.00E-72	329
WTH001_0012_N12	*Ferritin*	*Fer*	*Daphnia pulex*	ABK91576	2.00E-56	329
WTH001_0014_G13	*Ferritin*	*Fer*	*Daphnia pulex*	ABK91576	3.00E-15	329
WTH001_0002_C15	*Ferritin 1-like protein A*	*Fer1HCH*	*Daphnia pulex*	ABK91577	1.00E-81	114
WTH001_0002_L18	*Gasp precursor*	*Gasp*	*Drosophila melanogaster*	AAD09748	9.00E-21	910
WTH001_0005_P05	*Glutathione peroxidase*	*GPX*	*Ixodes scapularis*	AAY66814	2.00E-37	875
IGU001_0046_C04	*Glycogen phosphorylase*	*GlyP*	*Drosophila melanogaster*	NP_722762	5.00E-76	
IGU001_0032_H11	*GM2 ganglioside activator protein*	*GM2A*	***Xenopus laevis***	AAH74424	7.00E-07	587
WTH001_0010_I05	*GM2 ganglioside activator protein*	*GM2A*	***Danio rerio***	AAH92784	2.00E-07	587
WTH001_0012_G09	*GM2 ganglioside activator protein*	*GM2A*	***Danio rerio***	AAH92784	2.00E-07	587
WTH001_0005_E02	*Gram-negative bacteria binding protein*	*GNBP*	*Nasutitermes comatus*	AAZ08486	1.00E-17	74
WTH001_0007_G12	*Haemoglobin 1*	*dmHb1*	*Daphnia magna*	U67067	0.00E+00	391
IGU001_0009_B08	*Haemoglobin 2*	*dmHb2*	*Daphnia magna*	AB021136	2.00E-159	119
WTH001_0010_F04	*Haemoglobin 2*	*dmHb2*	*Daphnia magna*	AB021136	2.00E-122	119
WTH001_0012_E08	*Haemoglobin 2*	*dmHb2*	*Daphnia magna*	AB021136	2.00E-70	119
WTH001_0014_O13	*Haemoglobin 2*	*dmHb2*	*Daphnia magna*	AB021136	9.00E-79	119
WTH001_0004_I13	*Heat Shock Protein 20*	*HSP20*	*Locusta migratoria*	ABC84493	1.00E-40	414
R04CDF4A09	*Heat Shock Protein 70*	*HSP70*	***Bos taurus***	1NGH	2.00E-09	
WTH001_0007_N09	*Histone H4*	*H4*	***Mus musculus***	AAI15447	5.00E-21	
WTH001_0006_D22	*Innexin 2*	*Inx2*	*Bombyx mori*	AAR97567	3.00E-146	357
IGU001_0025_B06	*Insect cuticle protein (Chitin_bind_4)*	*ChtBD4*	*Tribolium castaneum*	XP_969336	2.00E-08	24
WTH001_0010_L07	*Insect pheromone-binding protein A10*	*A10*	*Apis mellifera*	CAJ01445	4.00E-14	131
WTH001_0005_H06	*Juvenile hormone esterase*	*JHE*	*Harmonia axyridis*	BAE16975	4.00E-11	
WTH001_0007_O20	*Lipoprotein N-terminal Domain (VTG related)*	*LPD_N*	*Aedes aegypti*	AB037697	1.00E-24	850
WTH001_0009_D09	*Lipoprotein N-terminal Domain (VTG related)*	*LPD_N*	*Aedes aegypti*	AB037697	1.00E-24	850
WTH001_0009_L11	*Lipoprotein N-terminal Domain (VTG related)*	*LPD_N*	*Aedes aegypti*	AB037697	1.00E-24	850
WTH001_0010_C02	*Lipoprotein N-terminal Domain (VTG related)*	*LPD_N*	*Aedes aegypti*	AB037697	1.00E-24	850

WTH001_0010_G23	*Lipoprotein N-terminal Domain (VTG related)*	*LPD_N*	*Aedes aegypti*	AB037697	1.00E-24	850
IGU001_0051_C02	*Myosin regulatory light chain 2*	*MRLC2*	*Gryllotalpa orientalis*	AAW22542	2.00E-40	1099
WTH001_0001_M24	*Na+/K+ ATPase alpha subunit*	*AT1A*	*Artemia franciscana*	CAA39972	4.00E-63	871
WTH001_0011_E12	*NADH dehydrogenase subunit 4*	*ND4*	*Daphnia pulex*	ABD19378	8.00E-22	425
IGU001_0001_H03	*Pleiotrophin*	*PTN*	*Apis mellifera*	XP_623430	2.00E-12	21
WTH001_0014_E10	*Polyubiquitin*	*UBQ*	***Bos taurus***	BAC56573	9.00E-53	
R14PHR1E10	*Protein disulfide isomerase*	*PDI*	*Tribolium castaneum*	XP_975184	1.00E-78	
WTH001_0004_H19	*RAS-like GTP-binding protein*	*RhoA*	*Aedes aegypti*	EAT34635	5.00E-60	546
IGU001_0014_F05	*Receptor expression-enhancing protein 5*	*REEP5*	***Danio rerio***	AAH59545	3.00E-41	
AD2C06	*Reelin*	*Reln*	*Lonomia obliqua*	AAV91350	8.00E-08	339
WTH001_0010_M21	*Ribosomal protein L14*	*RpL14*	*Apis mellifera*	XP_392809	5.00E-33	858
IGU001_0017_C06	*Ribosomal protein L15*	*RpL15*	*Ixodes ricinus*	AAY66921	2.00E-50	667
R01CDF1C01	*Ribosomal protein L22*	*RpL22*	*Tribolium castaneum*	XP_970247	9.00E-34	
IGU001_0031_B09	*Ribosomal protein L27*	*RpL27*	*Spodoptera frugiperda*	AAK92163	1.00E-35	260
WTH001_0003_A23	*Ribosomal protein L28*	*RpL28*	*Sphaerius sp.*	CAJ17404	1.00E-10	446
WTH001_0010_B19	*Ribosomal protein L30*	*RpL30*	*Argopecten irradians*	AF526198	4.00E-54	1260
WTH001_0014_L20	*Ribosomal protein L38*	*RpL38*	*Plutella xylostella*	BAD26684	4.00E-29	111
IGU001_0015_C06	*Ribosomal protein L6*	*RpL6*	*Carabus granulatus*	CAJ17232	5.00E-14	1031
IGU001_0030_G11	*Ribosomal protein L9*	*RpL9*	*Artemia franciscana*	ABC02755	7.00E-07	490
WTH001_0002_J09	*Ribosomal protein S10*	*RpS10*	*Ixodes scapularis*	AAY66831	3.00E-40	861
IGU001_0037_H02	*Ribosomal protein S12*	*RpS12*	*Periplaneta americana*	AAM33784	1.00E-24	704
R15LFF1H05	*Ribosomal protein S13*	*RpS13*	***Mus musculus***	XP_001004217	4.00E-25	
IGU001_0006_D10	*Ribosomal protein S17*	*RpS17*	*Diaphorina citri*	ABG81965	3.00E-41	88
IGU001_0036_D07	*Ribosomal protein S2*	*RpS2*	*Urechis caupo*	P49154	1.00E-59	1007
IGU001_0038_H10	*Ribosomal protein S20*	*RpS20*	***Oncorhynchus mykiss***	CAC44156	1.00E-50	84
IGU001_0056_E06	*Ribosomal protein S25*	*RpS25*	*Platystomos albinus*	CAH04344	6.00E-30	496
WTH001_0003_M18	*Ribosomal protein S3A*	*RpS3A*	*Tribolium castaneum*	XP_968064	8.00E-107	212
WTH001_0009_J03	*Ribosomal protein S3A*	*RpS3A*	*Culicoides sonorensis*	AAU06483	6.00E-85	212
IGU001_0031_E09	*Ribosomal protein S4*	*RpS4*	*Sphaerius sp.*	CAJ17169	7.00E-55	979
WTH001_0001_L14	*Rieske iron-sulfur protein*	*RISP*	*Lagothrix lagotricha*	Q69BJ8	7.00E-14	503
IGU001_0009_C10	*Selenoprotein 15*	*Sep15*	*Anopheles gambiae*	AAL68777	1.00E-35	
WTH001_0007_K22	*Serine collagenase 1 precursor*	*SC1*	*Celuca pugilator*	AAC47030	4.00E-07	358
WTH001_0012_E22	*Serine collagenase 1 precursor*	*SC1*	***Xenopus laevis***	AAH54215	5.00E-05	358
WTH001_0013_P02	*Serine collagenase 1 precursor*	*SC1*	*Celuca pugilator*	AAC47030	3.00E-49	358
WTH001_0013_L21	*Serine protease*	*SP*	*Pacifastacus leniusculus*	AAX55746	4.00E-07	
RRD03	*Serine-type protease inhibitor*	*Spint*	*Boophilus microplus*	CAC82583	5.00E-05	
IGU001_0016_F07	*S-phase kinase-associated protein 1*	*Skp1*	*Apis mellifera*	XP_392758	2.00E-30	1367
IGU001_0015_A05	*Sulfate transporter*	*Sfat*	*Aedes aegypti*	EAT35825	1.00E-27	
WTH001_0013_B11	*Sulfotransferase 1C2*	*Sult1C*	*Apis mellifera*	XP_394850	1.00E-30	716
WTH001_0001_I09	*Triacylglycerol lipase*	*Lip*	*Aedes aegypti*	EAT35159	2.00E-09	848

WTH001_0003_G14	*Triacylglycerol lipase*	*Lip*	*Antheraea yamamai*	BAD22559	1.00E-14	1039
IGU001_0052_C06	*Tropomyosin 1*	*Tm1*	*Charybdis feriatus*	Q9N2R3	2.00E-40	110
WTH001_0001_B03	*Trypsin*	*Try*	*Astacus astacus*	P00765	7.00E-29	
WTH001_0003_I06	*Trypsin*	*Try*	*Astacus astacus*	P00765	7.00E-29	919
WTH001_0003_I15	*Trypsin*	*Try*	*Aplysina fistularis*	AAO12215	1.00E-26	
WTH001_0005_C22	*Trypsin*	*Try*	*Aplysina fistularis*	AAO12215	2.00E-13	
WTH001_0006_C16	*Trypsin*	*Try*	*Artemia franciscana*	AAD39513	2.10E-55	919
WTH001_0006_G13	*Trypsin*	*Try*	*Aplysina fistularis*	AAO12215	3.00E-34	936
WTH001_0007_C09	*Trypsin*	*Try*	*Aplysina fistularis*	AAO12215	3.00E-57	919
WTH001_0007_E05	*Trypsin*	*Try*	*Astacus astacus*	P00765	6.00E-31	919
WTH001_0007_J17	*Trypsin*	*Try*	*Aplysina fistularis*	AAO12215	2.00E-13	
WTH001_0007_P01	*Trypsin*	*Try*	*Aplysina fistularis*	AAO12215	3.00E-57	919
WTH001_0009_C12	*Trypsin*	*Try*	*Aplysina fistularis*	AAO12215	3.00E-57	919
WTH001_0010_I08	*Trypsin*	*Try*	*Aplysina fistularis*	AAO12215	3.00E-57	
WTH001_0011_B16	*Trypsin*	*Try*	*Aplysina fistularis*	AAO12215	1.00E-45	
WTH001_0014_I05	*Trypsin*	*Try*	*Aplysina fistularis*	AAO12215	3.00E-57	919
WTH001_0014_K17	*Trypsin*	*Try*	*Aplysina fistularis*	AAO12215	3.00E-57	919
WTH001_0014_L15	*Trypsin*	*Try*	*Aplysina fistularis*	AAO12215	3.00E-57	919
WTH001_0012_B07	*Ubinuclein*	*Ubn*	***Homo sapiens***	AF108460	1.00E-05	412
IGU001_0006_G04	*Ubiquitin-like/S30 ribosomal fusion protein*	*RpS30*	*Lysiphlebus testaceipes*	AAX62410	4.00E-29	186
IGU001_0036_B03	*Vitelline membrane outer layer protein 1*	*VMO1*	***Canis familiaris***	XP_546575	6.00E-25	396
IGU001_0035_B07	*Vitellogenin 1*	*dmagVTG1*	*Daphnia magna*	BAD05137	1.00E-71	72
IGU001_0037_A07	*Vitellogenin 1*	*dmagVTG1*	*Daphnia magna*	BAD05137	2.00E-89	
WTH001_0006_J12	*Voltage-dependent anion-selective channel protein 2*	*VDAC2*	***Gallus gallus***	AAF73513	1.00E-61	1363
WTH001_0003_C17	*Voltage-gated Ca channel-related*	*VGCa*	*Tribolium castaneum*	XP_967004	8.00E-28	699
WTH001_0001_L02	*Zinc finger AN1-type*	*Znf_AN1*	*Apis mellifera*	XP_001121310	7.00E-09	1170
WTH001_0009_N03	Unknown					68
WTH001_0011_B21	Unknown					80
WTH001_0012_G15	Unknown					95
WTH001_0009_A18	Unknown					165
WTH001_0009_D07	Unknown					295
WTH001_0009_K20	Unknown					324
WTH001_0009_N07	Unknown					726
WTH001_0010_E16	Unknown					726
WTH001_0010_G07	Unknown					726
WTH001_0010_O02	Unknown					726
WTH001_0011_G05	Unknown					865
WTH001_0011_I19	Unknown					888
WTH001_0013_K18	Unknown					975
WTH001_0007_B09	Unknown					1237

IGU001_0002_G07	Unknown
IGU001_0003_H10	Unknown
IGU001_0004_D12	Unknown
IGU001_0004_E05	Unknown
IGU001_0009_A08	Unknown
IGU001_0009_B02	Unknown
IGU001_0009_F02	Unknown
IGU001_0010_H09	Unknown
IGU001_0011_F07	Unknown
IGU001_0015_D01	Unknown
IGU001_0023_B07	Unknown
IGU001_0024_H08	Unknown
IGU001_0025_E06	Unknown
IGU001_0026_F04	Unknown
IGU001_0027_A05	Unknown
IGU001_0027_C01	Unknown
IGU001_0027_C11	Unknown
IGU001_0028_D10	Unknown
IGU001_0029_E02	Unknown
IGU001_0030_G09	Unknown
IGU001_0031_A02	Unknown
IGU001_0031_E02	Unknown
IGU001_0033_E06	Unknown
IGU001_0034_B07	Unknown
IGU001_0034_E10	Unknown
IGU001_0035_B08	Unknown
IGU001_0037_B09	Unknown
IGU001_0037_F08	Unknown
IGU001_0041_D10	Unknown
IGU001_0042_A08	Unknown
IGU001_0042_D04	Unknown
IGU001_0044_F02	Unknown
IGU001_0047_D10	Unknown
IGU001_0047_E12	Unknown
IGU001_0047_F03	Unknown
IGU001_0047_G11	Unknown
IGU001_0048_B09	Unknown
IGU001_0048_F05	Unknown
IGU001_0049_E12	Unknown
IGU001_0050_D03	Unknown

IGU001_0050_G10	Unknown
IGU001_0051_C06	Unknown
IGU001_0052_A02	Unknown
IGU001_0052_D12	Unknown
IGU001_0052_E08	Unknown
IGU001_0055_D11	Unknown
R01CDF1B02	Unknown
R01CDF1F03	Unknown
R08CDR4B08	Unknown
R15LFF1A02	Unknown
RAA06	Unknown
WTH001__0014_I07	Unknown
WTH001_0001_C23	Unknown
WTH001_0001_J03	Unknown
WTH001_0001_M03	Unknown
WTH001_0002_D02	Unknown
WTH001_0002_E06	Unknown
WTH001_0002_G23	Unknown
WTH001_0002_I16	Unknown
WTH001_0003_K14	Unknown
WTH001_0003_L13	Unknown
WTH001_0003_O05	Unknown
WTH001_0004_E01	Unknown
WTH001_0004_H21	Unknown
WTH001_0004_J01	Unknown
WTH001_0005_D02	Unknown
WTH001_0005_L14	Unknown
WTH001_0006_A14	Unknown
WTH001_0006_E21	Unknown
WTH001_0006_F04	Unknown
WTH001_0006_K12	Unknown
WTH001_0007_A03	Unknown
WTH001_0012_M07	Unknown
WTH001_0013_N24	Unknown
WTH001_0013_O23	Unknown

[a]Sequences for clone ID IGU001_XXXX_XXX and WTH001_XXXX_XXX are available on DaphniaBase [55]. RXXXXXXXXX clones are available on the website of the *Daphnia* research group of the University of Reading [56]. Clone ID AD2C06 is published on GenBank [52] under accession no. EH669342; [b]Non-invertebrate species matches are indicated in bold font; [c]Contig sequences are available on DaphniaBase [55]. Note that some genes match more than one contig, however multiple contig sequences match similar BLAST annotations.
