# Supplementary material for: Systems biology meets stress ecology: linking molecular and organismal stress responses in Daphnia magna
Source: Genome Biol. 2008 Feb 21;9(2):R40. doi: 10.1186/gb-2008-9-2-r40 (PMC2374704; doi:10.1186/gb-2008-9-2-r40)
Supplement: Additional data file 2 — Presented is a table showing the relative expression and GO of the unique D. magna genes responding to acute ibuprofen exposure. [file gb-2008-9-2-r40-S2.pdf]

Additional data file 2. Differentially expressed *Daphnia magna* genes following acute exposure to ibuprofen (IB)*.

Putative gene	20 mg IB l^{-1}	40 mg IB l^{-1}	80 mg IB l^{-1}	GO#	GO description
**1. METABOLISM**					
5'-nucleotidase-related	1.122	0.503	0.355	GO:0009435	NAD biosynthesis
Acyl CoA synthetase	0.736	0.543	0.430	GO:0004467	long-chain-fatty-acid-CoA ligase activity
ADP/ATP translocase	0.607	0.484	0.258	GO:0005471	ATP:ADP antiporter activity
Alpha amylase	1.223	1.355	1.588	GO:0004556	alpha-amylase activity
Astacin	0.927	0.794	0.419	GO:0008533	astacin activity (metalloendopeptidase activity)
ATP synthase a chain[a]	1.114	1.142	0.972	GO:0015986	ATP synthesis coupled proton transport
Bovine pancreatic trypsin inhibitor domain	1.327	1.363	1.725	GO:0004867	serine-type endopeptidase inhibitor activity
Carboxypeptidase A2	1.362	1.499	0.688	GO:0004182	carboxypeptidase A activity
Cathepsin L-like protease precursor[a]	1.515	1.455	1.734	GO:0004217	cathepsin L activity (proteolysis)
Chitinase[a]	1.255	1.541	2.043	GO:0006036	cuticle chitin catabolism
Chymotrypsin B2[a]	0.956	1.253	1.137	GO:0004263	chymotrypsin activity (proteolysis)
Cytochrome b[a]	1.354	1.454	0.880	GO:0006122	mitochondrial electron transport, ubiquinol to cytochrome c
Cytochrome c oxidase subunit 1[a]	1.216	0.988	0.920	GO:0006123	mitochondrial electron transport, cytochrome c to oxygen
Cytochrome c oxidase subunit 2[a]	0.639	0.494	0.573	GO:0006123	mitochondrial electron transport, cytochrome c to oxygen
Endo-beta-1,4-mannanase	2.428	3.046	1.529	GO:0004553	hydrolase activity, hydrolyzing O-glycosyl compounds
Enolase[a]	2.343	2.236	1.024	GO:0004634	phosphopyruvate hydratase activity (glycolysis)
Glutathione peroxidase	0.729	0.251	0.889	GO:0004602	glutathione peroxidase activity
Glycogen phosphorylase	1.901	1.044	0.509	GO:0005980	glycogen catabolism
GM2 ganglioside activator protein[a]	1.635	1.765	1.219	GO:0016004	phospholipase activator activity
NADH dehydrogenase subunit 4	1.206	0.344	0.293	GO:0008137	NADH dehydrogenase (ubiquinone) activity
Leukotriene B_4 12-hydroxydehydrogenase[b]	1.192	1.587	3.502	GO:0006691	leukotriene metabolic process
Rieske iron-sulfur protein	1.478	1.758	0.980	GO:0006118	electron transport
Serine collagenase 1 precursor[a]	1.612	1.887	1.555	GO:0006508	proteolysis
Serine protease	0.664	0.463	0.557	GO:0006508	proteolysis
Serine-type protease inhibitor	0.841	0.655	0.523	GO:0006508	proteolysis
Sulfotransferase 1C2	1.558	1.339	0.589	GO:0008146	sulfotransferase activity
Triacylglycerol lipase[a]	1.307	1.502	2.410	GO:0004806	triacylglycerol lipase activity
Trypsin[a]	1.561	1.692	1.358	GO:0006508	proteolysis
**2. GENETIC INFORMATION PROCESSING**					
16S ribosomal RNA[a]	1.018	1.128	1.155	GO:0003735	structural constituent of ribosome
28S ribosomal RNA[a]	1.398	1.529	1.227	GO:0005842	cytosolic large ribosomal subunit (sensu Eukaryota)
DEAD Box ATP-dependent RNA helicase	1.845	1.147	3.591	GO:0000184	mRNA catabolism, nonsense-mediated decay
Heat Shock Protein 20	1.901	2.195	2.848	GO:0006986	response to unfolded protein
Histone H4	0.575	0.523	0.453	GO:0006334	nucleosome assembly
Polyubiquitin	2.328	2.221	7.229	GO:0000209	protein polyubiquitination
Protein disulfide isomerase	1.035	0.646	0.846	GO:0006457	protein folding
Ribosomal protein L14	1.442	1.626	1.030	GO:0006412	translation

Ribosomal protein L15	1.076	1.362	1.558	GO:0006412	translation
Ribosomal protein L22	0.678	0.664	0.718	GO:0006412	translation
Ribosomal protein L27	0.481	0.276	0.0925	GO:0006412	translation
Ribosomal protein L28	1.646	1.904	0.923	GO:0006412	translation
Ribosomal protein L30	1.382	2.003	1.917	GO:0006412	translation
Ribosomal protein L38	1.755	2.559	1.469	GO:0006412	translation
Ribosomal protein L6	3.063	1.611	0.814	GO:0006412	translation
Ribosomal protein L9	0.866	0.518	0.362	GO:0006412	translation
Ribosomal protein S10	0.981	0.698	1.140	GO:0006412	translation
Ribosomal protein S12	1.408	1.540	2.125	GO:0006412	translation
Ribosomal protein S13	0.648	0.795	0.705	GO:0006412	translation
Ribosomal protein S17	0.928	0.839	0.591	GO:0006412	translation
Ribosomal protein S2	1.414	1.269	2.347	GO:0006412	translation
Ribosomal protein S20	1.734	2.011	0.717	GO:0006412	translation
Ribosomal protein S25	1.664	1.951	1.178	GO:0006412	translation
Ribosomal protein S3A[a]	1.510	1.415	2.703	GO:0006412	translation
Ribosomal protein S4	1.396	1.526	2.133	GO:0006412	translation
Selenoprotein 15	5.031	5.960	2.462	GO:0051084	posttranslational protein folding
Ubinuclein	0.971	0.750	0.420	GO:0008134	transcription factor binding
Ubiquitin-like/S30 ribosomal fusion protein	1.640	1.658	1.829	GO:0006412	translation
**3. ENVIRONMENT INFORMATION PROCESSING**					
Contactin 1	1.223	0.823	0.246	GO:0007155	cell adhesion
C-terminal binding protein	1.311	0.556	0.684	GO:0008285	negative regulation of cell proliferation
Heat Shock Protein 70	1.928	2.542	1.650	GO:0006986	response to unfolded protein
Innexin 2	2.594	1.698	0.981	GO:0015286	innexin channel activity
Insect pheromone-binding protein A10	2.770	1.740	2.054	GO:0007606	sensory perception of chemical stimulus
Pleiotrophin	1.436	1.457	1.881	GO:0008083	growth factor activity
RAS-like GTP-binding protein	2.111	1.904	1.637	GO:0007266	Rho protein signal transduction
Receptor expression-enhancing protein 5	1.478	1.707	1.407	GO:0009401	phosphoenolpyruvate-dependent sugar phosphotransferase
S-phase kinase-associated protein 1	0.656	0.743	0.784	GO:0016567	protein ubiquitination
Voltage-dependent anion-selective channel protein 2	1.315	1.505	0.989	GO:0006820	anion transport
**4. CELLULAR PROCESSES**					
Actin[a]	1.138	0.776	0.654	GO:0005200	structural constituent of cytoskeleton
Calcification-associated peptide-2 precursor[a]	0.751	0.373	0.508	GO:0042302	structural constituent of cuticle
Chitin-binding domain type 2	2.93	1.394	0.964	GO:0008061	chitin binding
Chitin-binding Peritrophin-A domain	1.530	1.313	1.176	GO:0016490	structural constituent of peritrophic membrane (sensu Insecta)
C-type lectin-like	0.703	0.710	0.405	GO:0006954	inflammatory response
CUB domain	1.039	0.996	0.562	GO:0006954	inflammatory response
Cuticle protein 7[a]	0.823	0.428	0.447	GO:0042302	structural constituent of cuticle
Cuticle structural protein post-ecdysial PCP16.7	0.698	0.864	0.64	GO:0042302	structural constituent of cuticle

Cuticular protein precursor[a]	0.885	1.013	1.127	GO:0042302	structural constituent of cuticle
Cysteine-rich intestinal protein	2.217	1.192	0.452	GO:0008270	zinc ion binding
DD5	0.421	0.396	0.340	GO:0042302	structural constituent of cuticle
Fatty acid binding protein 3	1.000	1.434	1.430	GO:0005504	fatty acid binding
Ferritin[a]	1.280	1.460	1.952	GO:0006879	iron ion homeostasis
Ferritin 1-like protein A	1.915	1.866	1.716	GO:0006879	iron ion homeostasis
Gasp precursor	4.301	2.742	1.541	GO:0016490	structural constituent of peritrophic membrane (sensu Insecta)
Gram-negative bacteria binding protein	0.902	0.786	0.505	GO:0045088	regulation of innate immune response
Haemoglobin 1	0.884	0.402	0.456	GO:0015671	oxygen transport
Haemoglobin 2[a]	1.475	1.024	0.970	GO:0015671	oxygen transport
Insect cuticle protein (Chitin_bind_4)	0.694	0.884	0.575	GO:0042302	structural constituent of cuticle
Juvenile hormone esterase	0.528	0.218	0.102	GO:0006719	juvenile hormone catabolism
Lipoprotein N-terminal Domain (VTG related)[a]	0.825	0.533	0.707	GO:0005319	lipid transporter activity
Myosin regulatory light chain 2	1.717	0.983	1.786	GO:0006940	regulation of smooth muscle contraction
Na+/K+ ATPase alpha subunit	1.410	1.332	1.234	GO:0005391	sodium:potassium-exchanging ATPase activity
Reelin	1.309	1.486	1.336	GO:0000904	cellular morphogenesis during differentiation
Sulfate transporter	0.424	0.278	0.351	GO:0008272	sulfate transport
Tropomyosin 1	1.698	1.673	1.281	GO:0006936	muscle contraction
Vitelline membrane outer layer protein 1	0.713	0.812	0.863	GO:0009993	oogenesis (sensu Insecta)
Vitellogenin 1[a]	1.083	1.143	1.723	GO:0007296	vitellogenesis
Voltage-gated Ca channel-related	3.713	3.245	3.760	GO:0006812	cation transport
Zinc finger AN1-type	1.425	0.731	0.877	GO:0008270	zinc ion binding

*Genes have been categorised based on their gene ontology (GO) annotation. Exposed expression levels are shown relative to controls; [a]Expression data of redundant genes, aligned in the same sequence contig or with identical annotations, are shown as averages; [b]*Leukotriene B_4 12-hydroxydehydrogenase* (*Ltb4dh*) responded on the microarray, but the fluorescent emission levels were below the set detection criteria. However, previously QPCR data validate a concentration-dependent induction of *Ltb4dh* (see Heckmann et al., 2006 [21]).
