# Supplementary material for: Systems biology meets stress ecology: linking molecular and organismal stress responses in Daphnia magna
Source: Genome Biol. 2008 Feb 21;9(2):R40. doi: 10.1186/gb-2008-9-2-r40 (PMC2374704; doi:10.1186/gb-2008-9-2-r40)
Supplement: Additional data file 3 — Presented is a table displaying QPCR confirmation of selected D. magna genes responding on the cDNA microarray. [file gb-2008-9-2-r40-S3.pdf]

Additional data file 3. Real-time quantitative PCR (QPCR) confirmation of selected *Daphnia magna* genes responding on cDNA microarray following acute exposure to ibuprofen (IB)[a].

	0 mg IB l^{-1}	20 mg IB l^{-1}	40 mg IB l^{-1}	80 mg IB l^{-1}	R^2-values[b]	Primers (5'-3')[e]	Amplicon size (bp)	Amplification efficiency (E)[f]
C-type Lectin like (*CLECT*)[c]	1.000<br>1.000±0.081	0.703±0.336<br>0.812±0.062	0.710±0.296<br>0.695±0.017*	0.405±0.016<br>0.477±0.044*	0.92 (L)	TTCCACGTCGAAACCCTAAC<br>CCACTCCCGTCCAGTAGGTA	88	1.038±0.046
Glutathione Peroxidase (*GPX*)	1.000<br>1.000±0.043	0.729±5.156<br>0.862±0.012*	0.251±0.054<br>0.895±0.041	0.889±3.588<br>1.113±0.027	0.95 (C)	ACTTCCAGCCGAACATAACG<br>GAGTCGTCGGACAGCTTCTT	99	1.017±0.018
Juvenile Hormone Esterase (*JHE*)[c]	1.000<br>1.000±0.119	0.528±0.551<br>0.726±0.148	0.218±0.030<br>0.492±0.027*	0.102±0.178<br>0.173±0.028*	0.87 (L)	ATGGAGTTCTCAACGGAACG<br>ATCTCAGGTGTGGGCATTTC	100	0.896±0.016
Triacylglycerol Lipase (*Lip*)[cd]	1.000<br>1.000±0.044	1.307±0.189<br>2.129±0.599	1.502±0.194<br>2.757±0.101*	2.410±2.077<br>6.124±0.395*	1.00 (L)	CTGTTTATTGGCGACATTGC<br>TAGCCGTTCATTCTCCAACC	95	0.941±0.025
Ubinuclein (*Ubn*)	1.000<br>1.000±0.104	0.971±0.142<br>0.799±0.075	0.750±0.164<br>0.809±0.086	0.420±0.028<br>1.127±0.046	0.56 (C)	GGCACAGTGGTTTCCGTATC<br>TGCTTGTTCCGGTGTTACTG	81	1.033±0.022
Vitellogenin 1 (*DmagVTG1*)[cd]	1.000<br>1.000±0.104	1.083±0.526<br>0.984±0.049	1.143±0.410<br>1.034±0.088	1.723±1.502<br>1.232±0.065	0.96 (L)	CTGGCAAATGGGAAATCAAC<br>CCCAGGTGTAAGCCAAACC	93	0.870±0.084

[a]Microarray expression levels are shown in the first line and QPCR expression levels are shown below (mean ± SE, n = 3-4). The QPCR amplicons were quantified from *Daphnia* exposed in a comparable independent, previously reported (see Heckmann et al., 2006 [21]), experiment. QPCR expression was calculated using DART-PCR [59] and normalised to a geNorm [60] estimated normalisation factor based on the geometric mean of *actin*, *glyceraldehyde-3-phosphate dehydrogenase* and *ubiquitin conjugating enzyme*. Note that, although being differentially expressed on the microarray, *actin* has previously been verified as a QPCR reference gene [21]. Exposed expression levels are shown relative to controls; [b]R^2-values of correlated QPCR and microarray expression. Brackets signify a linear (L) or cubic (C) correlation; [c]Significant ($P < 0.05$, linear regression) concentration-dependent relationship between IB concentration and relative QPCR expression. Expression levels significant different ($P < 0.05$, Student's t-test) from controls are indicated by an asterisks (*); [d]Expression data of redundant microarray genes, aligned in the same sequence contig or with identical annotations, have been averaged; [e]Upper and lower sequences represent forward and reverse primers respectively; [f]Values are mean ± SD (N = 36).
