# Supplementary material for: Systems biology meets stress ecology: linking molecular and organismal stress responses in Daphnia magna
Source: Genome Biol. 2008 Feb 21;9(2):R40. doi: 10.1186/gb-2008-9-2-r40 (PMC2374704; doi:10.1186/gb-2008-9-2-r40)

Additional data file 4. *Daphnia magna* embryo developmental stage 1-2 (*sensu* Kast-Hutcheson et al., 2001 [30]). Embryogenesis was arrested within ~24 h during a 4-8 day maternal exposure to ibuprofen (80 mg l^{-1}). Note that arrested embryos were aborted at the same time as healthy neonates were released in the controls (see Table 2).

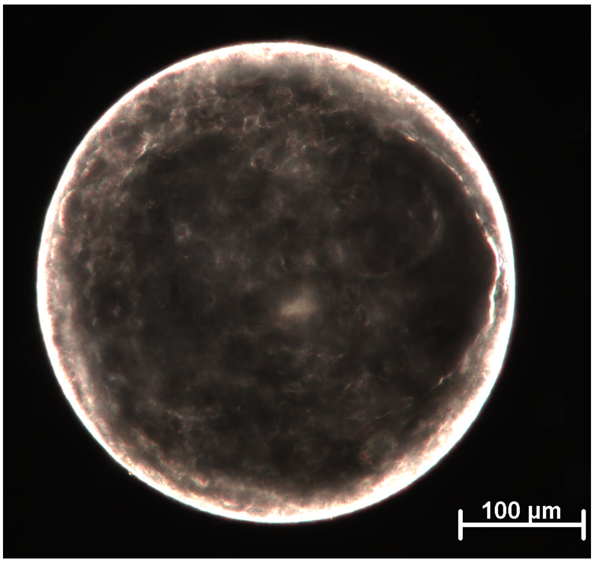

Supplement: Additional data file 4 — Presented is an image of a D. magna embryo arrested at developmental stage 1 to 2 after maternal exposure to ibuprofen. [file gb-2008-9-2-r40-S4.pdf]
