# Supplementary material for: Systems biology meets stress ecology: linking molecular and organismal stress responses in Daphnia magna
Source: Genome Biol. 2008 Feb 21;9(2):R40. doi: 10.1186/gb-2008-9-2-r40 (PMC2374704; doi:10.1186/gb-2008-9-2-r40)
Supplement: Additional data file 5 — Presented is a document with supplementary methods on microarray hybridization and microarray construction. [file gb-2008-9-2-r40-S5.pdf]

Additional data file 5. Supplementary methods on microarray hybridisation and microarray construction.

Microarray hybridisation

Pre-hybridisation washes

Following incubation in pre-hybridisation buffer (see Methods), slides were washed in ultra high pure water and 100% isopropanol and immediately dried by spinning in a Christ RVC 2-25 CD centrifuge (Martin Christ GmBH, Germany) for 2 min at 1,350 rpm.

Post-hybridisation washes

After 16 h of hybridisation (see Methods) slides were washed briefly in 2x SSC to remove the coverslip followed by 2 x 5 min washes in 0.1x SSC and 0.1% SDS, and 2 x 5 min washes in 0.1x SSC while being gently agitated (80 rpm) on a Stuart orbital shaker (Stuart Scientific Ltd., UK) at room temperature. Finally, the slides were washes once in 0.05x SSC, and once in 100% isopropanol and immediately dried by spinning in a Christ RVC 2-25 CD centrifuge (Martin Christ GmBH, Germany) for 2 min at 1,350 rpm.

Microarray construction

Construction of cDNA libraries

The *Daphnia magna* microarray was constructed using cDNA fragments from three sources: i) Stress-specific cDNAs were generated using suppression subtractive hybridisation (SSH) on organisms exposed to the five different stressors (ibuprofen, cadmium, lufenuron, pH and calcium). SSH was performed with forward and reverse subtraction using a PCR-Select cDNA Subtraction Kit (Clontech Laboratories Inc, UK). cDNAs (2,029 clones) were synthesised from mRNA extracted from batches of 100 *D. magna* (24-48 h old) using a Straight A's mRNA Isolation Kit System (Novagen Inc., UK). Because of a high abundance of ribosomal RNA still being present with the mRNA sample, post-synthesised cDNA was later amplified using a Super Smart PCR cDNA Synthesis Kit (Clontech Laboratories Inc., UK). This process relatively reduces the abundance of rRNA by amplifying the copy number of polyA synthesised product. cDNA fragments were then cloned with plasmid vectors pCR® 4-TOPO® into competent *Escherichia coli* cells using a TOPO TA Cloning Kit for Sequencing (Invitrogen, UK). Resulting colonies were amplified using M13-Long primers (forward 5' cgacgttgtaaaacgacggccag 3' and reverse 5' caggaaacagctatgaccatgattacgcc 3'). The amplified products were purified using Millipore Montage 96-well cleanup plates (VWR International, UK). ii) ESTs (10,272 clones) obtained from a cDNA library from unexposed mixed aged organisms obtained from a 3-4 week old culture [18]. iii) cDNAs isolated following an SSH (1,143 clones), as above, between 15 adults carrying eggs and 75 juveniles [7].

Microarray printing

Spots were pin-printed on Corning CMT-UltraGAPS glass slides (Fisher Scientific, UK) in a 17 x 18 block format with 48 blocks per microarray (grid = 14,688) using an Omnigrid 100 (Genomics Solutions, USA). Dimethyl sulphoxide (DMSO) was added, to a final concentration of 50%, to the purified PCR products prior to printing. A number of positive and negative hybridisation control spots were printed in each of the 48 blocks on the arrays. The positive hybridisation controls comprised of *D. magna* genomic DNA and four Spot Report System PCR products from *Arabidopsis thaliana*; *CAB*, *RCA*, *RBCL* and *LPT4* (Stratagene, USA). Negative hybridisation controls consisted of salmon sperm DNA, mouse *Cot-1*, human *Cot-1*, yeast tRNA and polyA RNA. Blank spots (50% DMSO) were interspaced with the controls and jointly used to qualitatively assess microarray hybridisation efficiency and provide orientation for the grid overlap during assessment. After printing, microarray slides were UV cross-linked (150mJ/cm^2) followed by baking at 80°C for 2 h. Slides were stored at room temperature under vacuum in total darkness.
