# Supplementary material for: Systems biology meets stress ecology: linking molecular and organismal stress responses in Daphnia magna
Source: Genome Biol. 2008 Feb 21;9(2):R40. doi: 10.1186/gb-2008-9-2-r40 (PMC2374704; doi:10.1186/gb-2008-9-2-r40)
Supplement: Additional data file 6 — Presented are MA plots of raw and normalized microarray data [file gb-2008-9-2-r40-S6.pdf]

Additional data file 6. MA plots of raw and normalised microarray data (ibuprofen-exposed *D. magna*)*

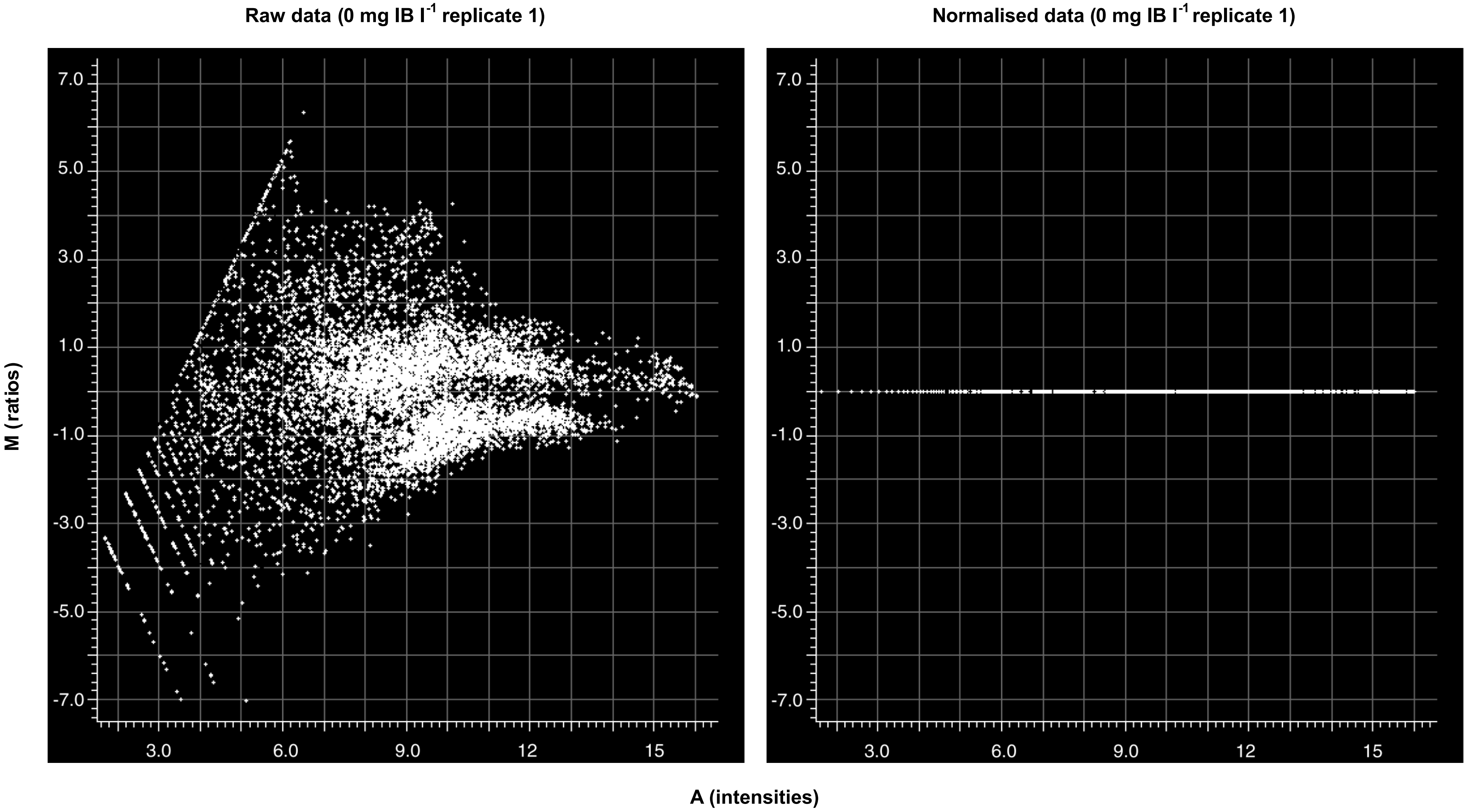

MA plots continued.

Raw data (20 mg IB l^{-1} replicate 1) **Normalised data (20 mg IB l^{-1} replicate 1)**

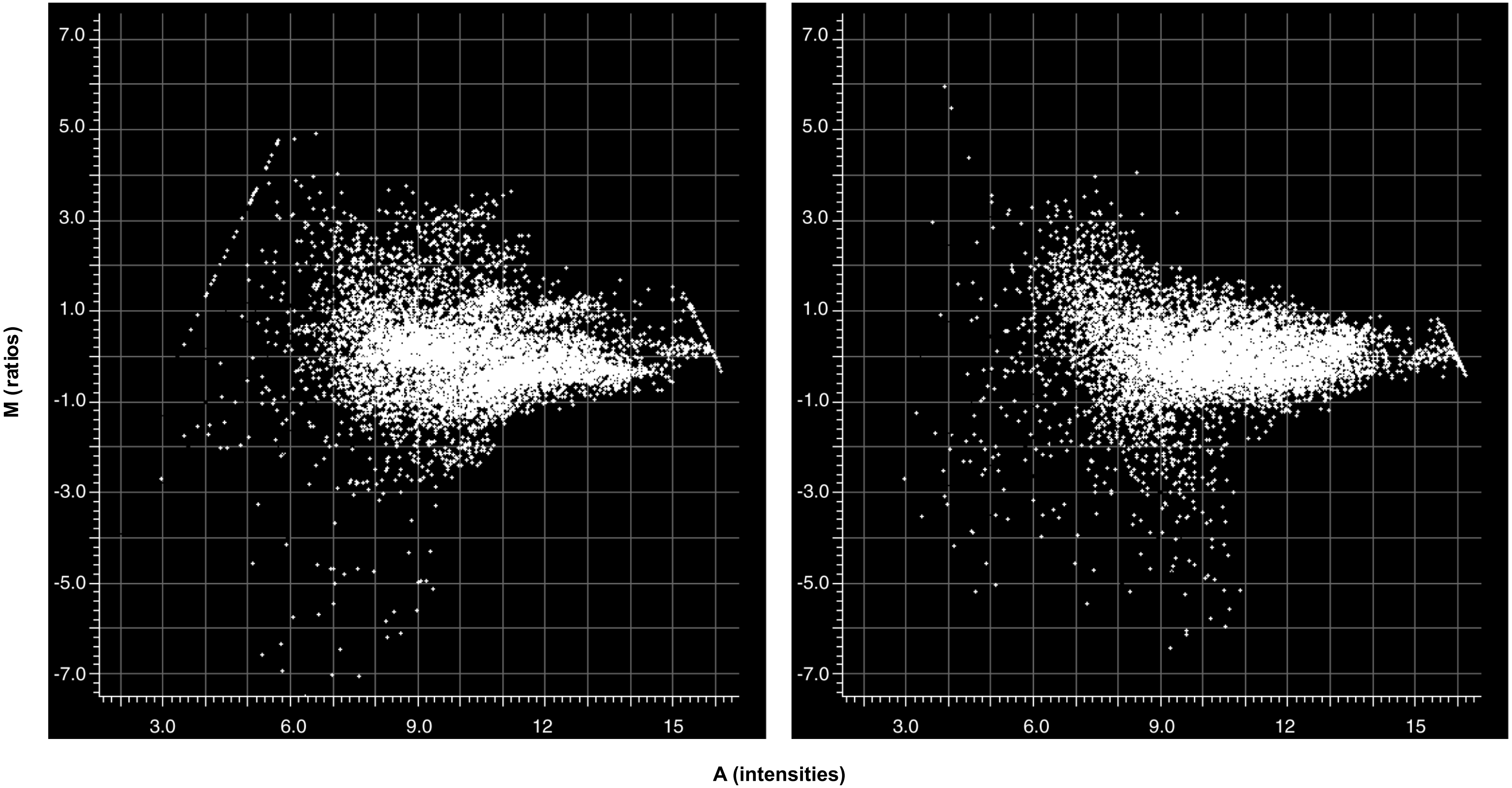

MA plots continued.

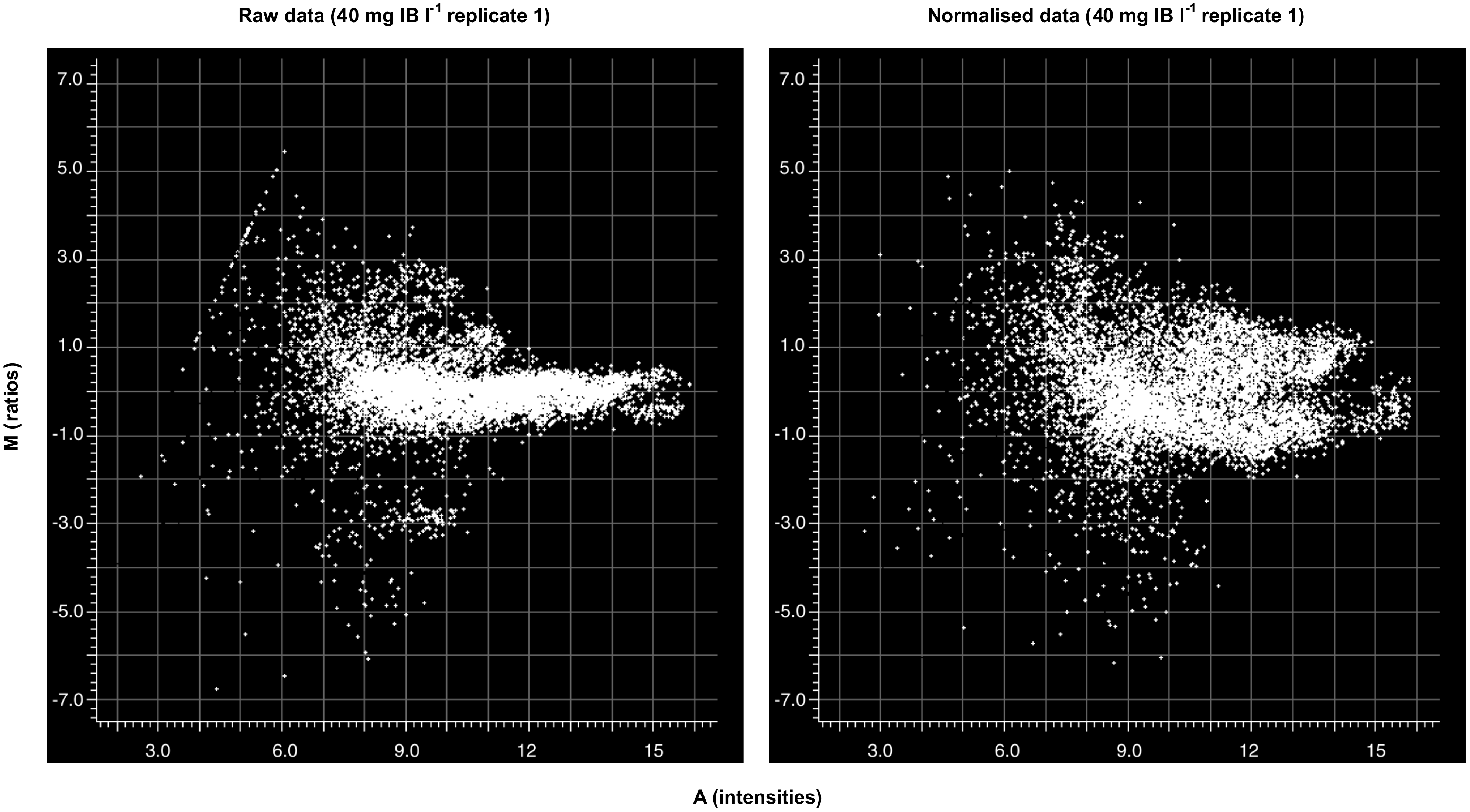

MA plots continued.

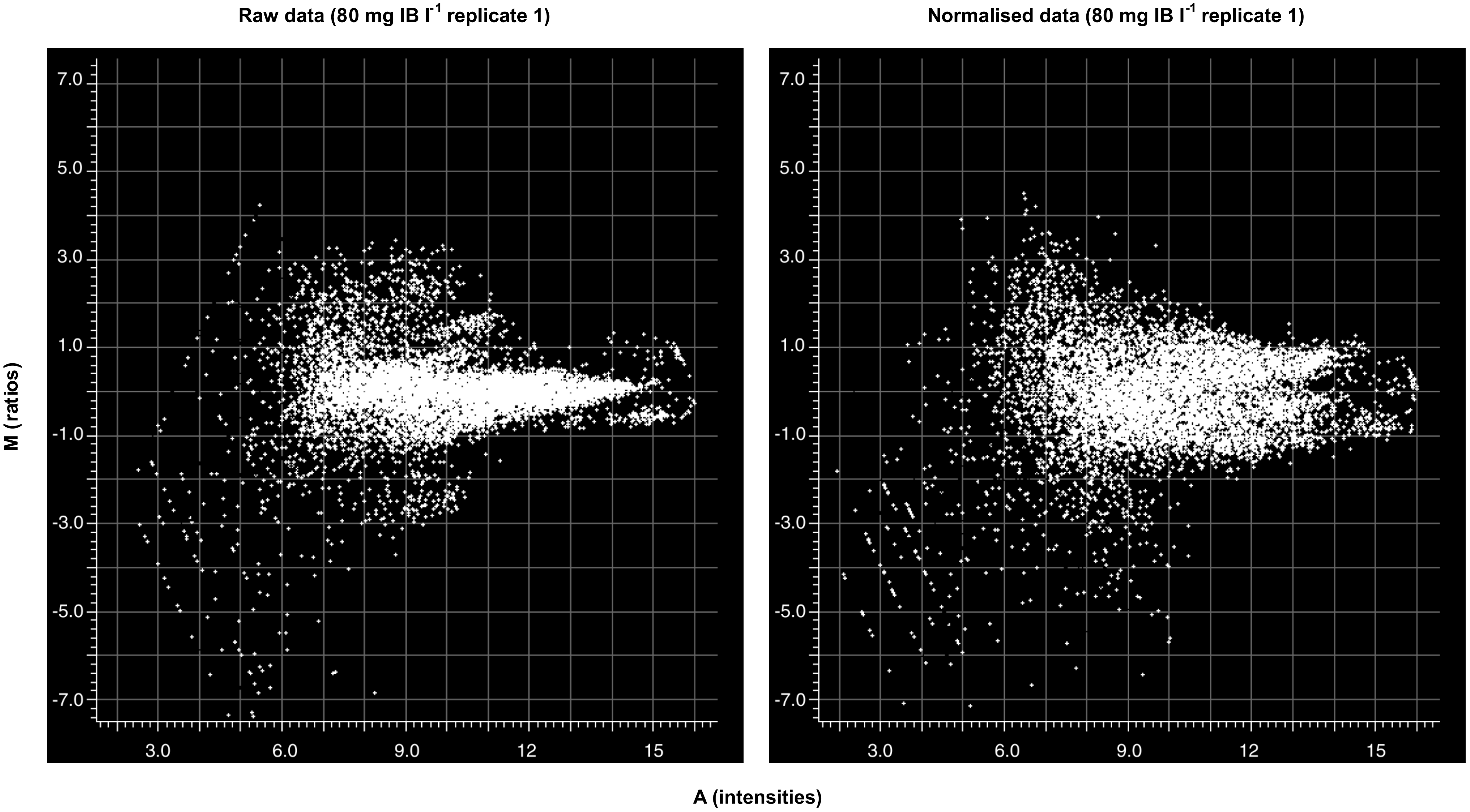

MA plots continued.

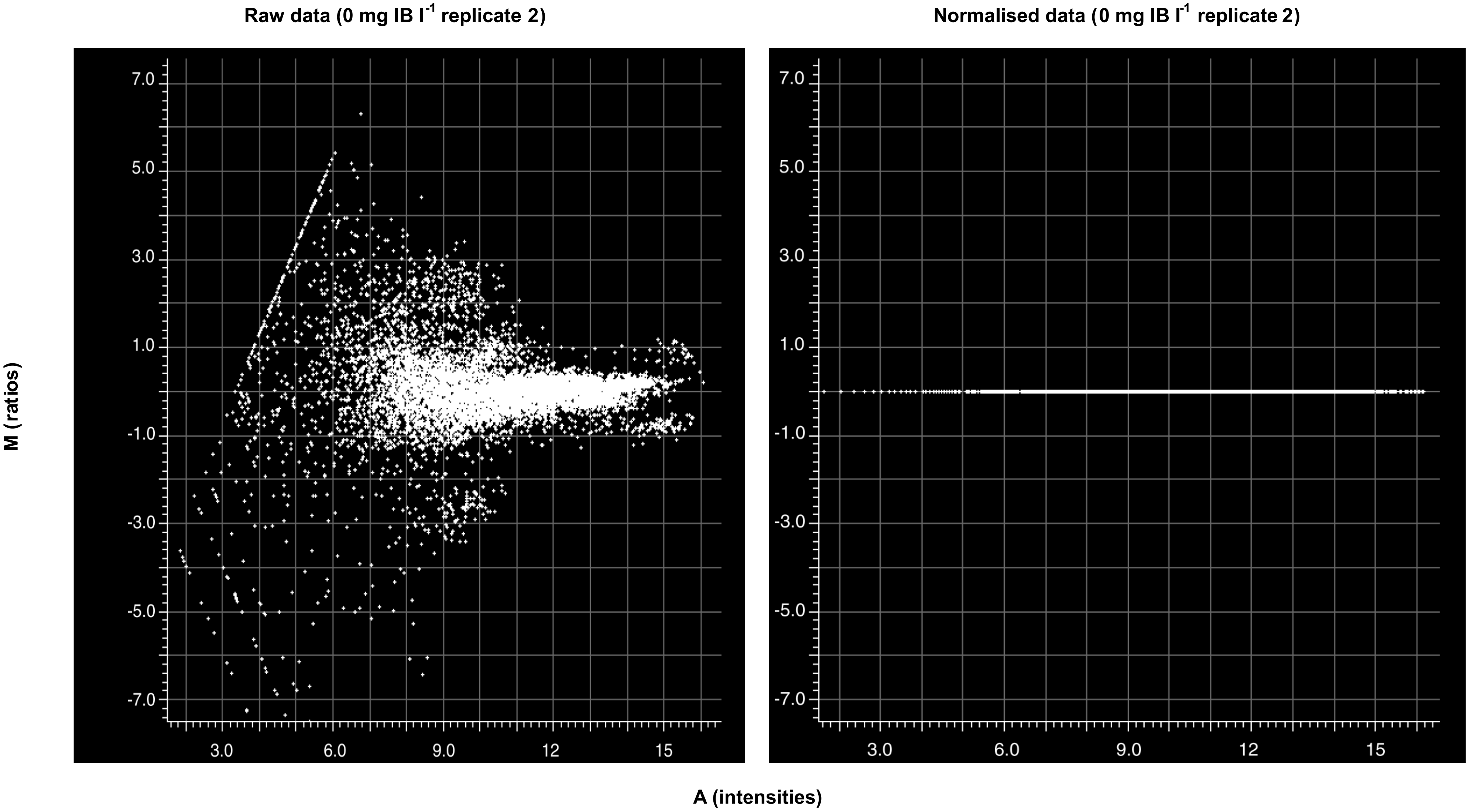

MA plots continued.

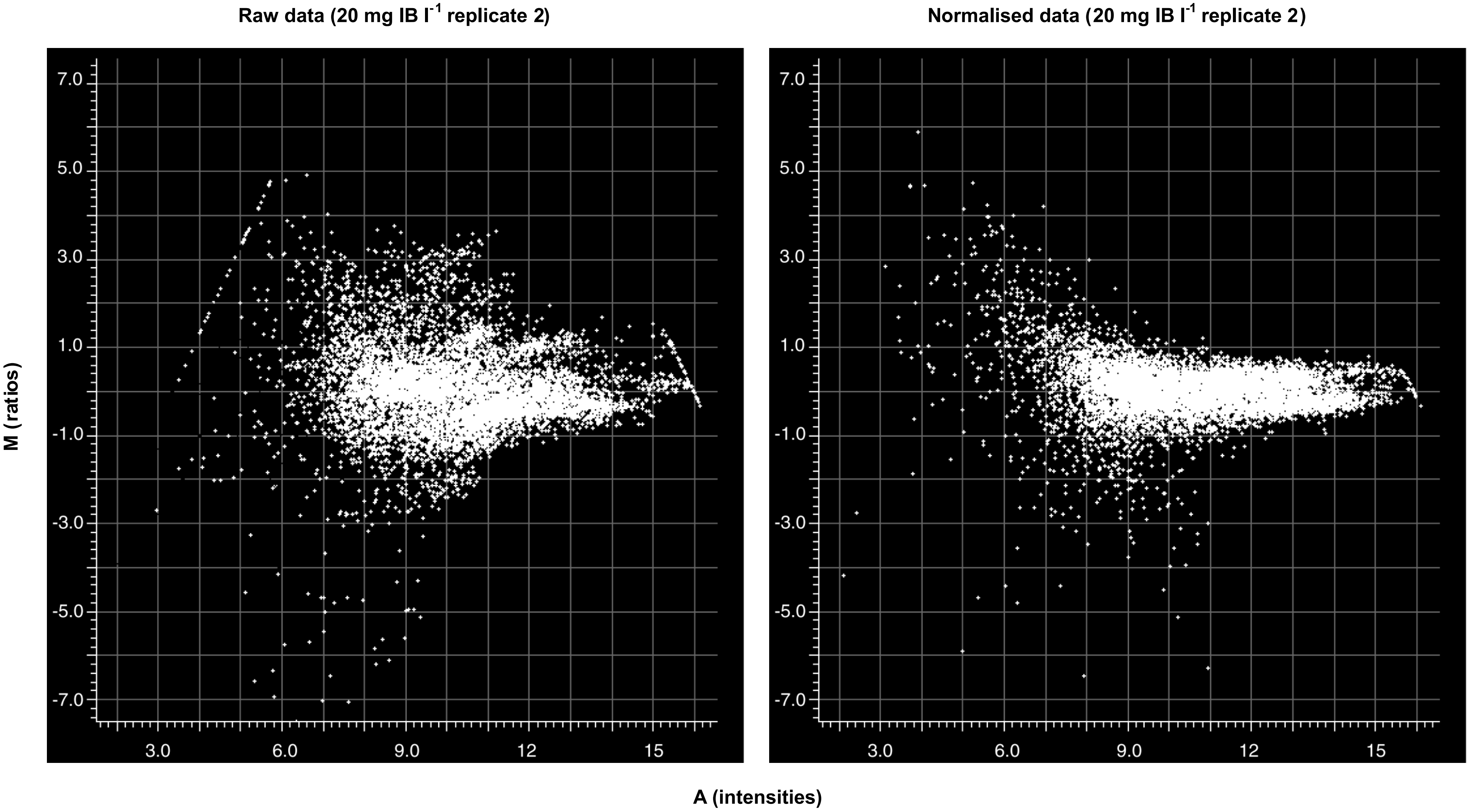

MA plots continued.

Raw data (40 mg IB l^{-1} replicate 2) **Normalised data (40 mg IB l^{-1} replicate 2)**

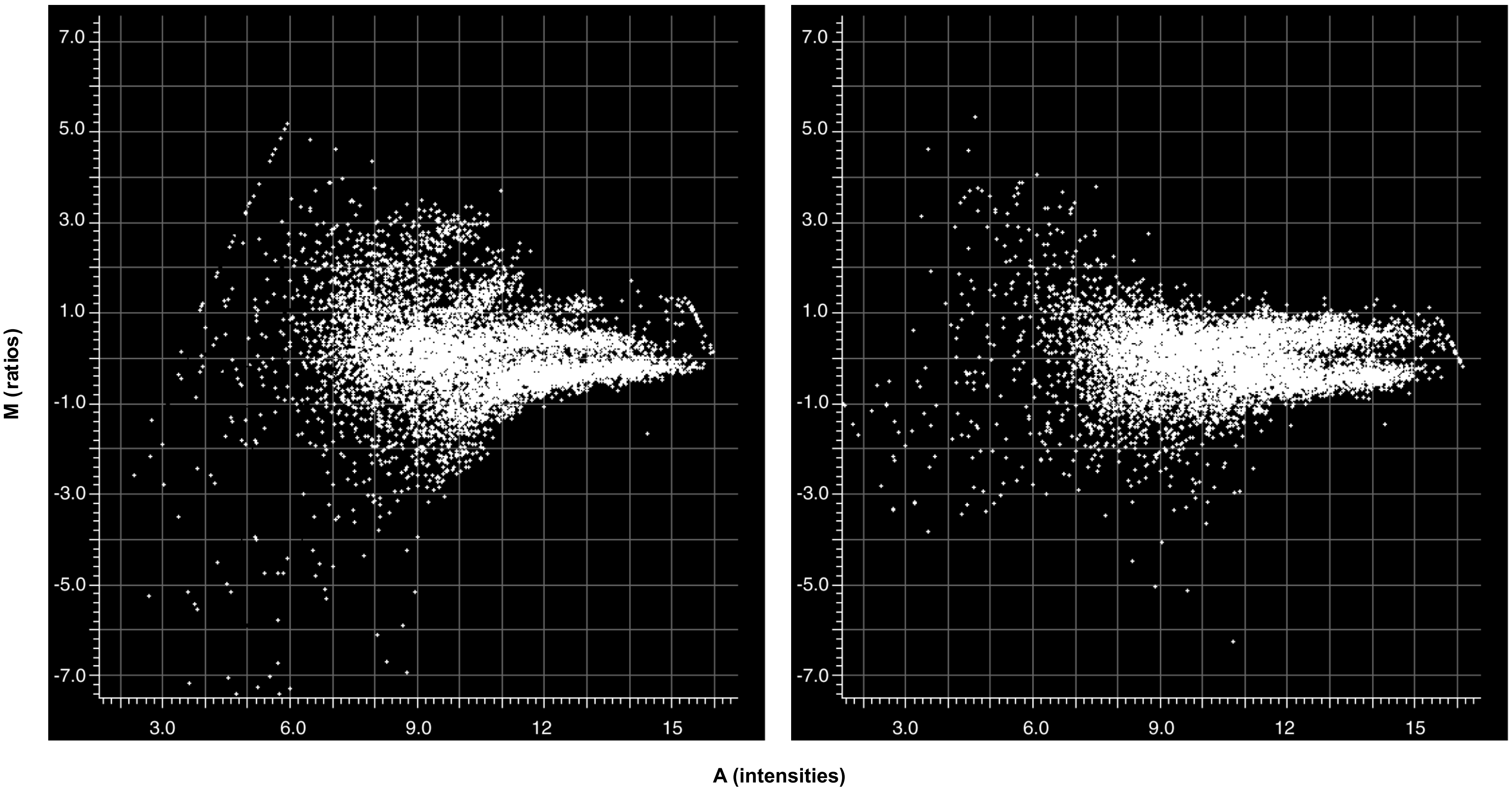

MA plots continued.

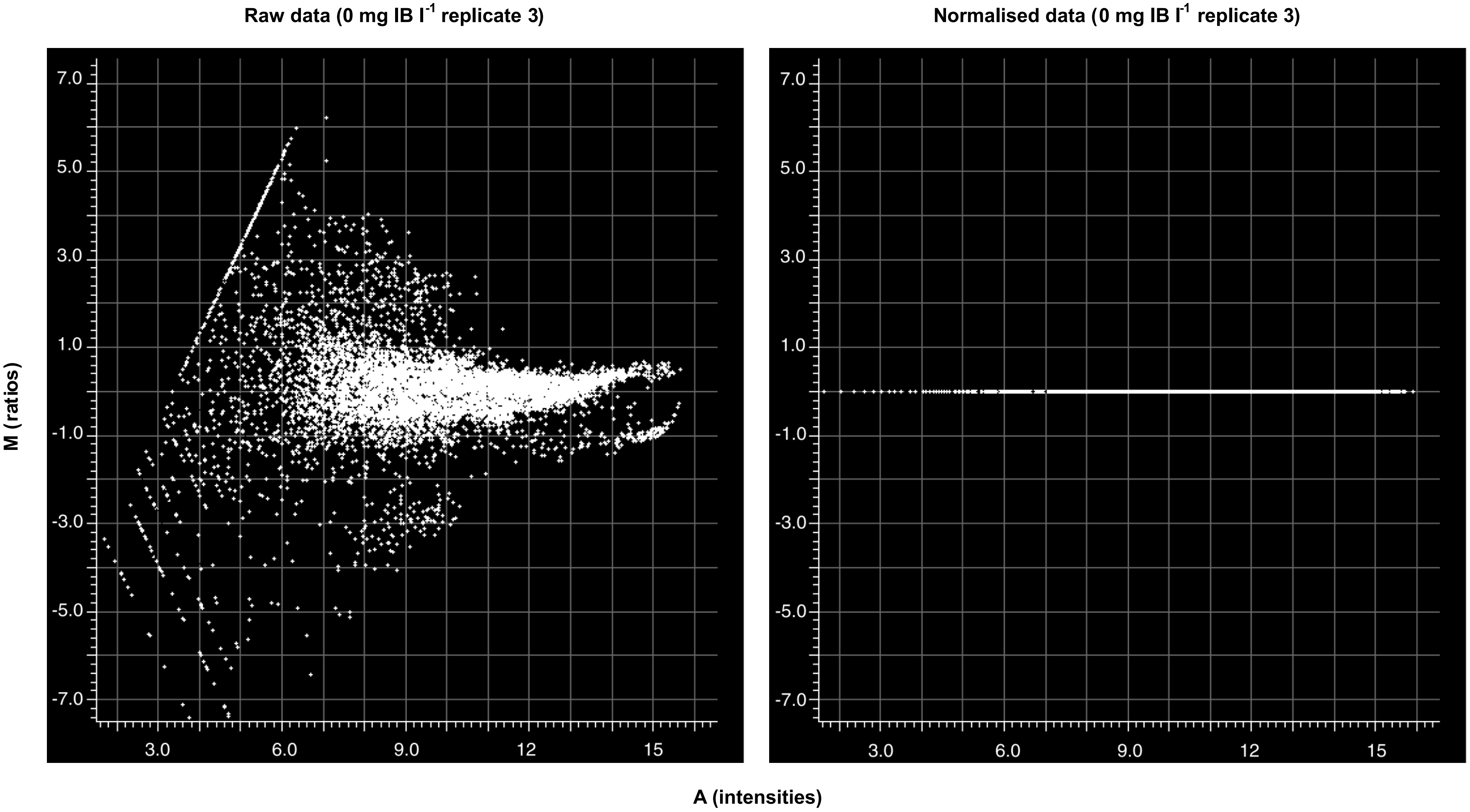

MA plots continued.

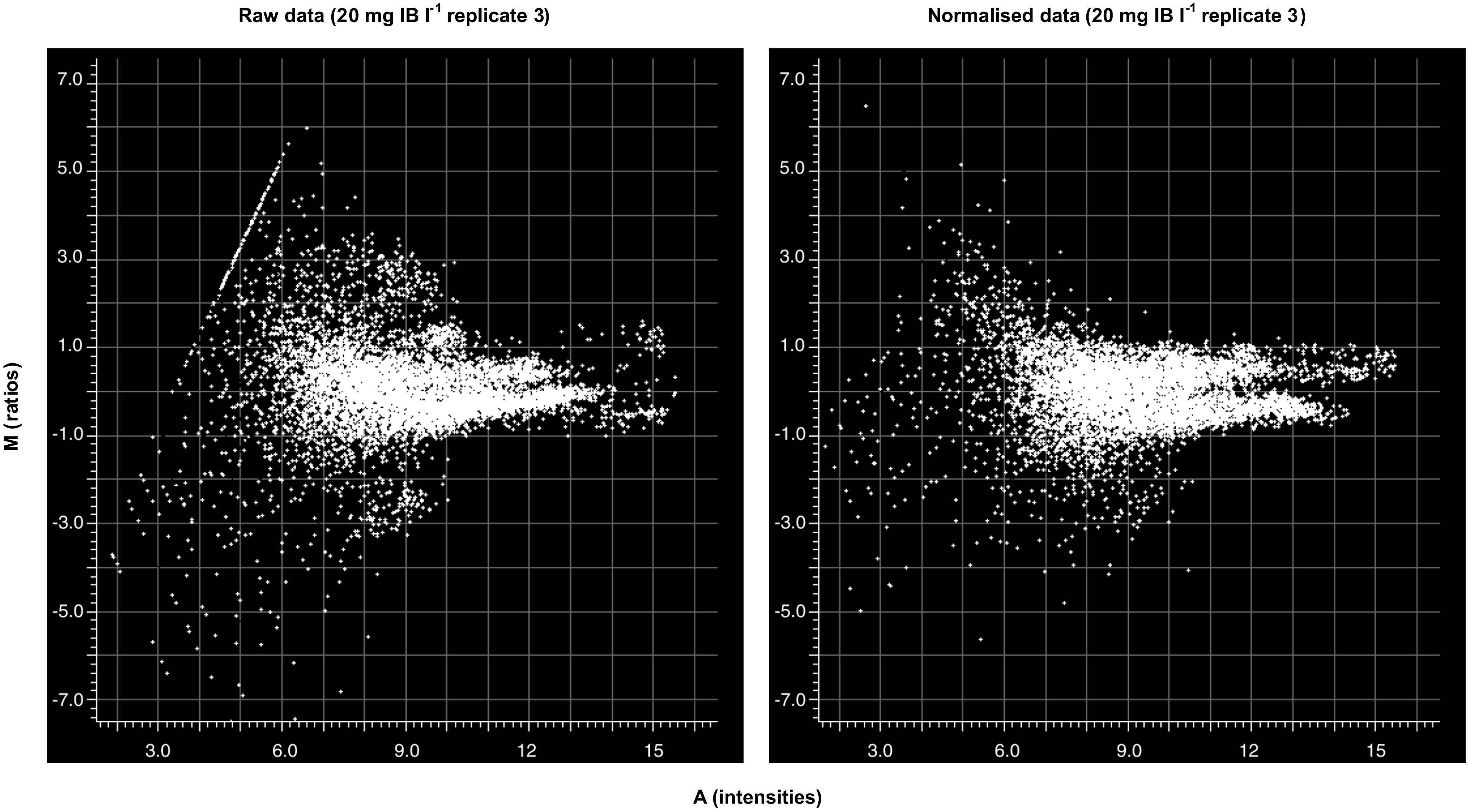

MA plots continued.

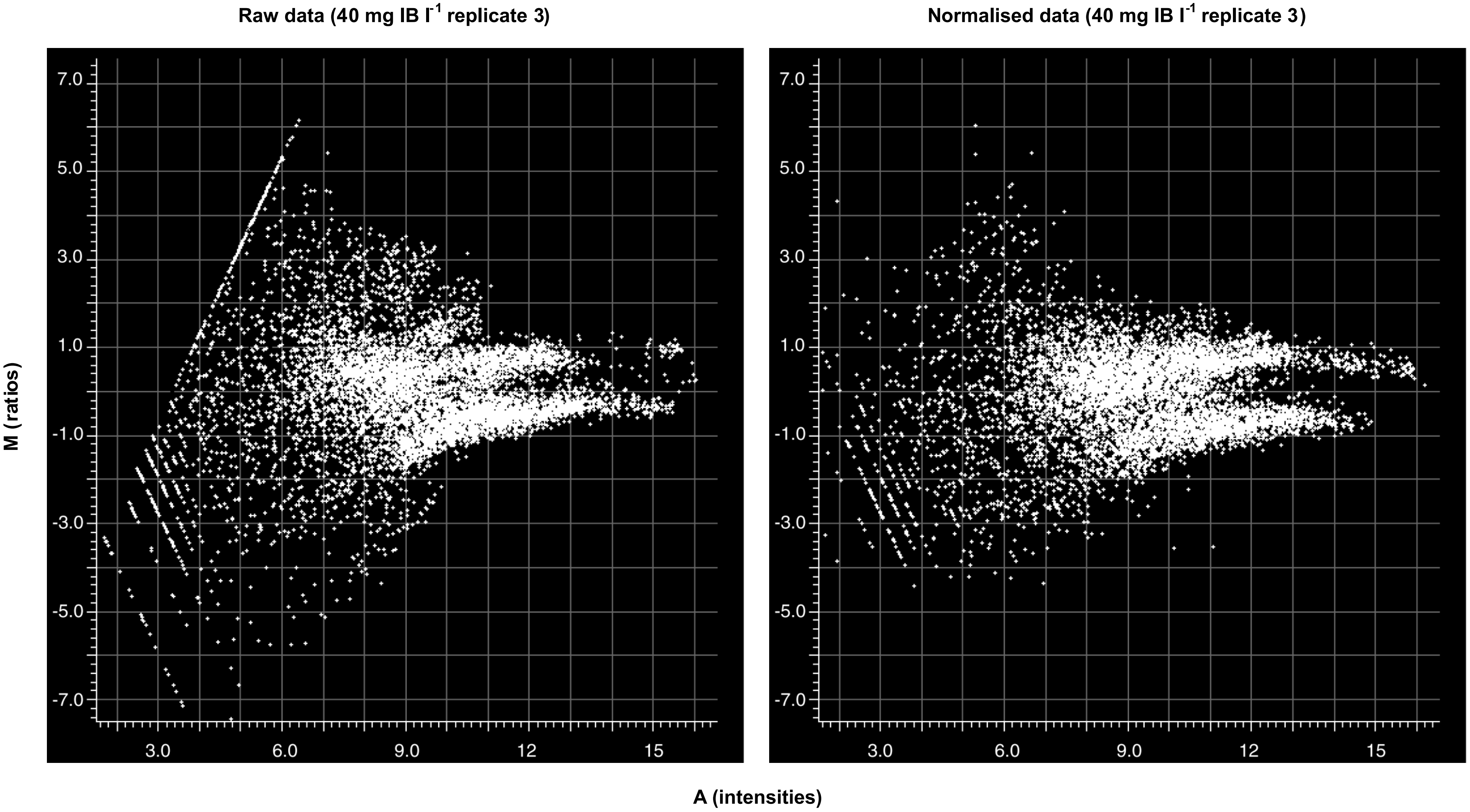

MA plots continued.

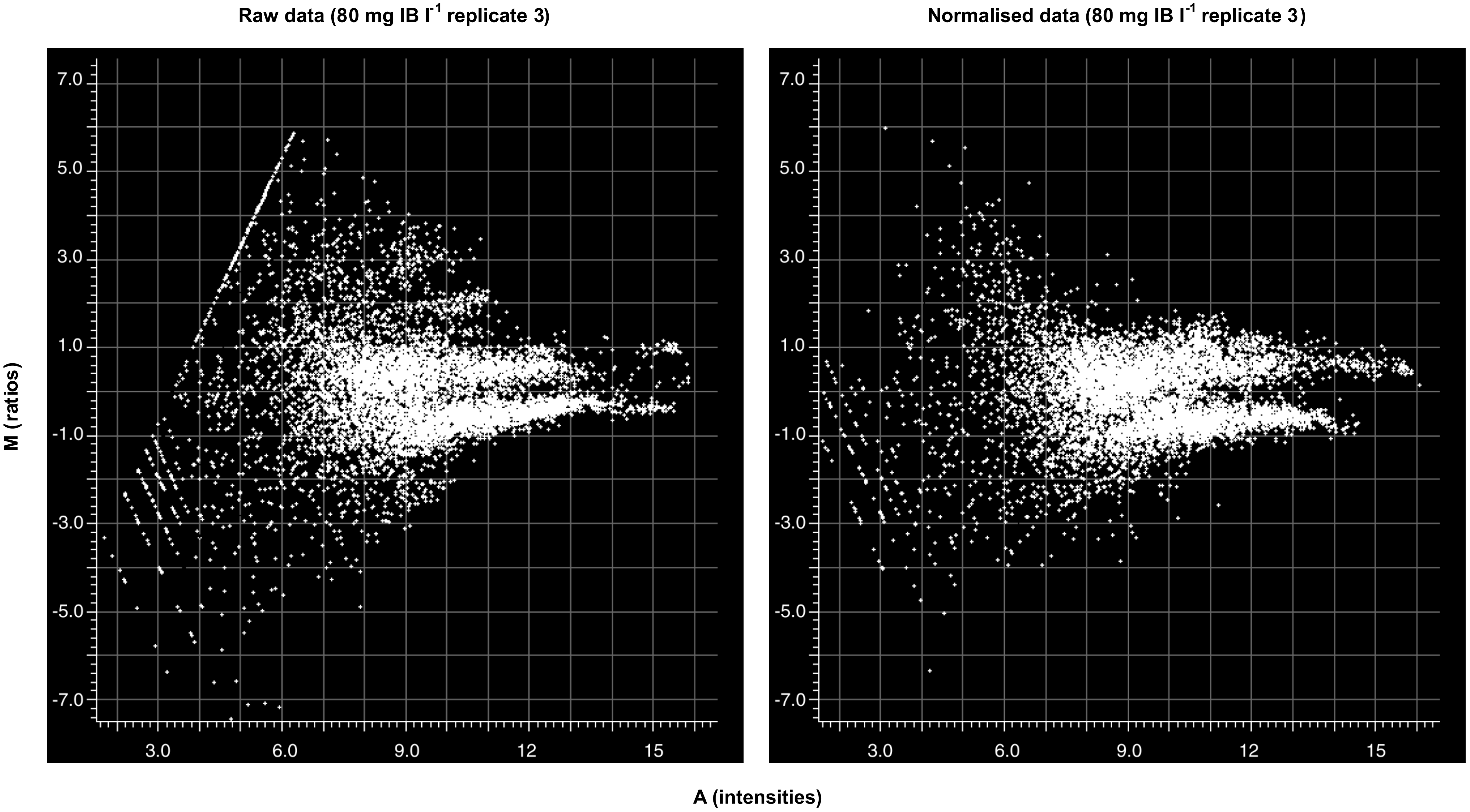

MA plots continued.

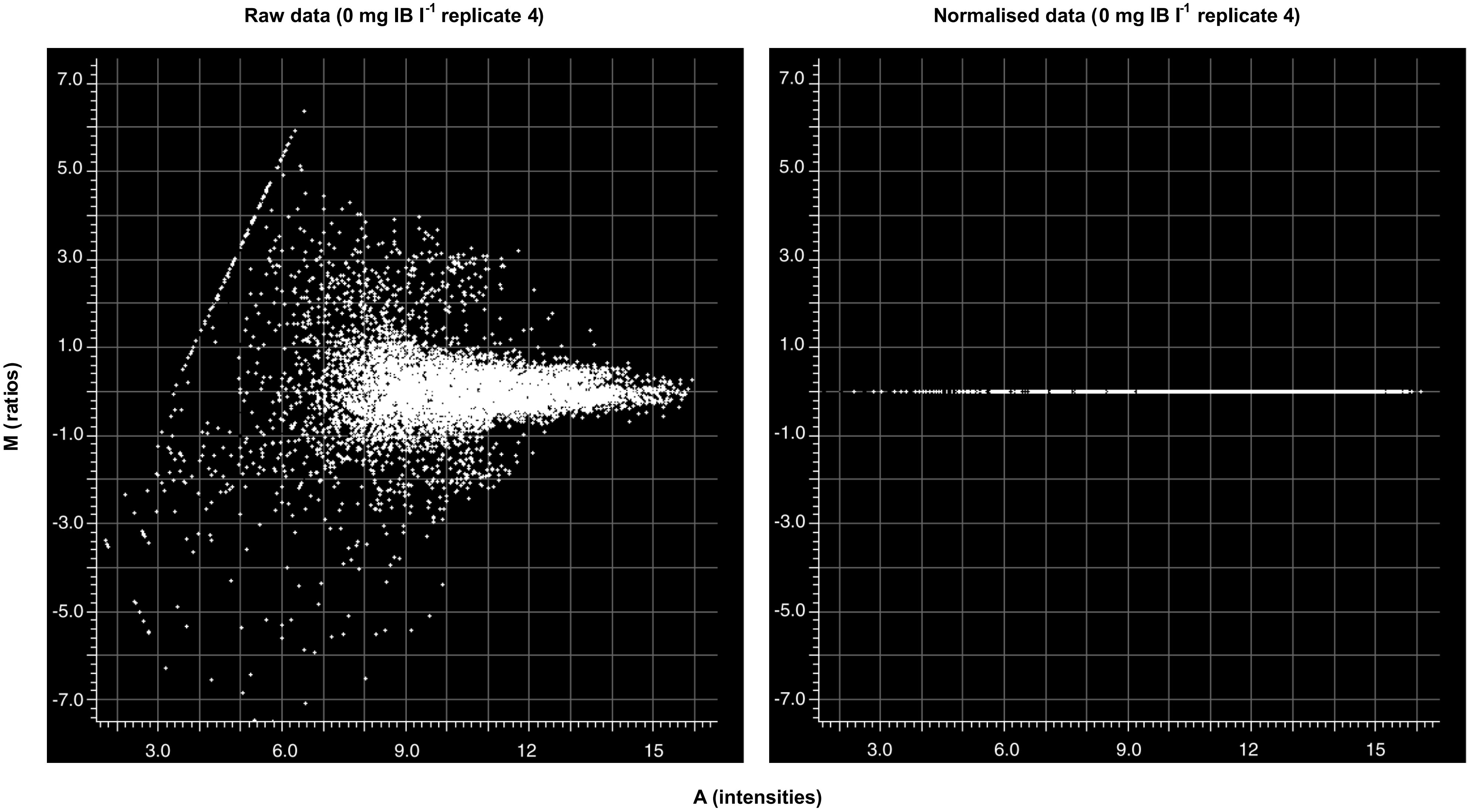

MA plots continued.

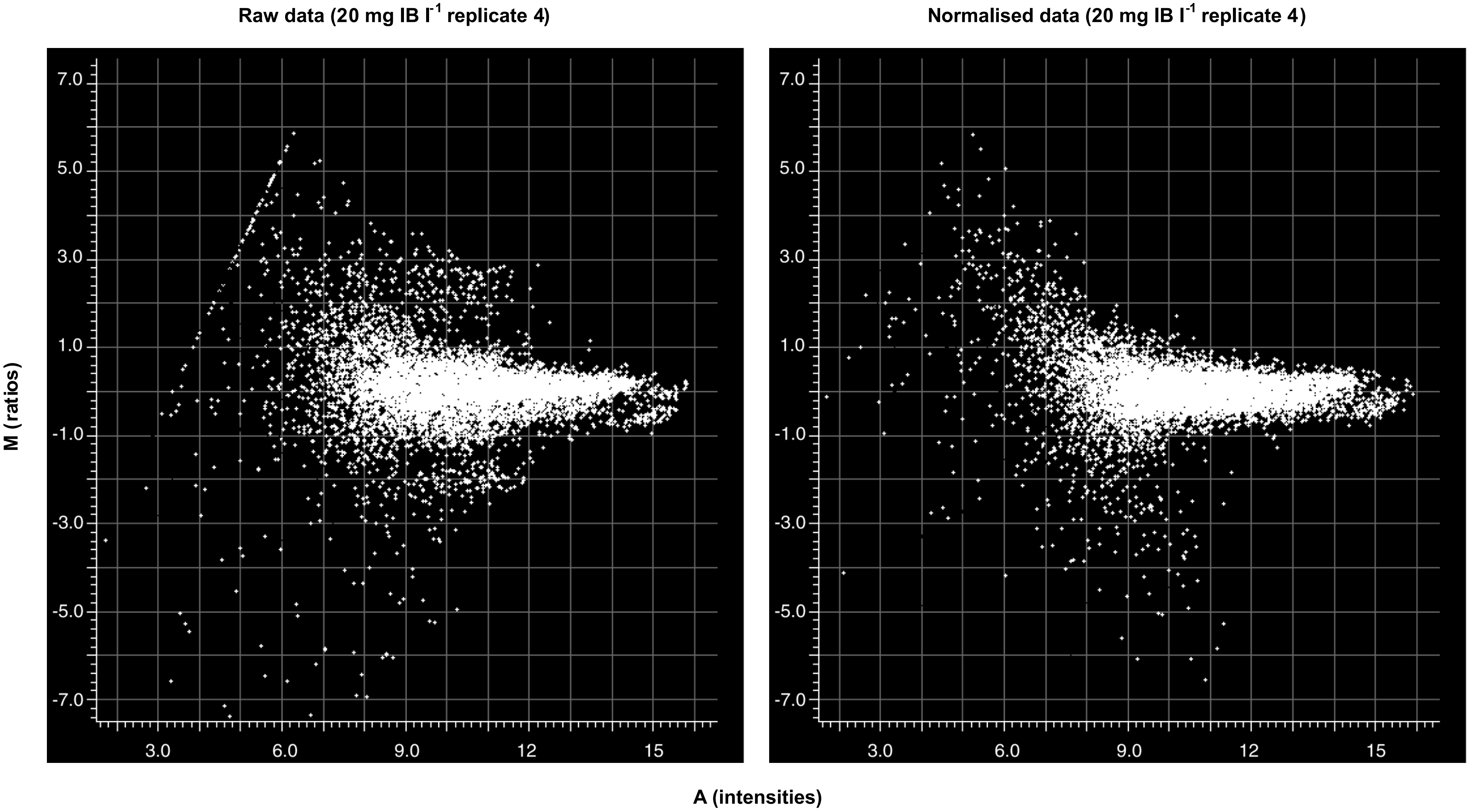

MA plots continued.

Raw data (40 mg IB l^{-1} replicate 4) **Normalised data (40 mg IB l^{-1} replicate 4)**

M (ratios)

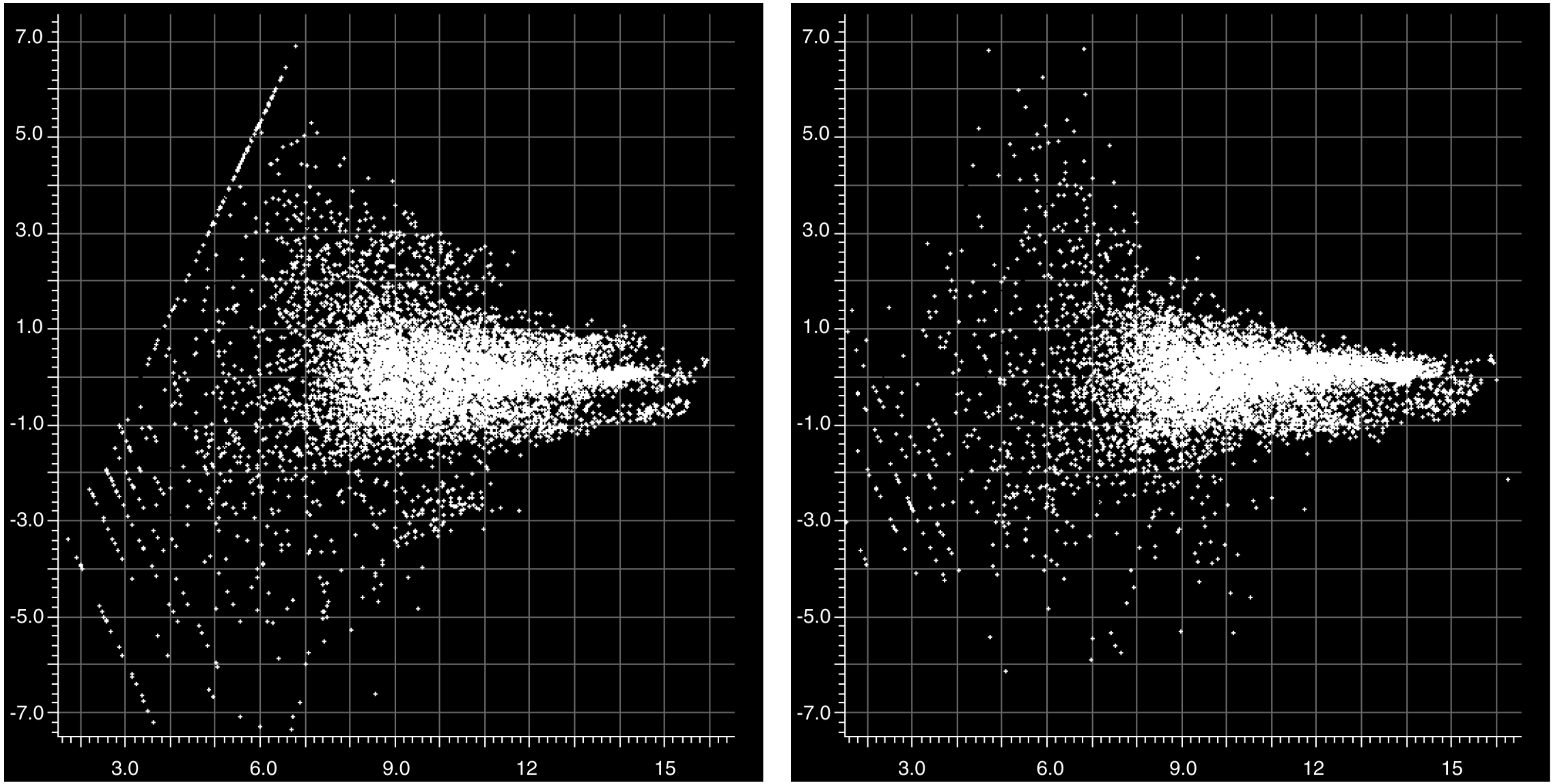

A (intensities)

MA plots continued.

Raw data (80 mg IB l^{-1} replicate 4)

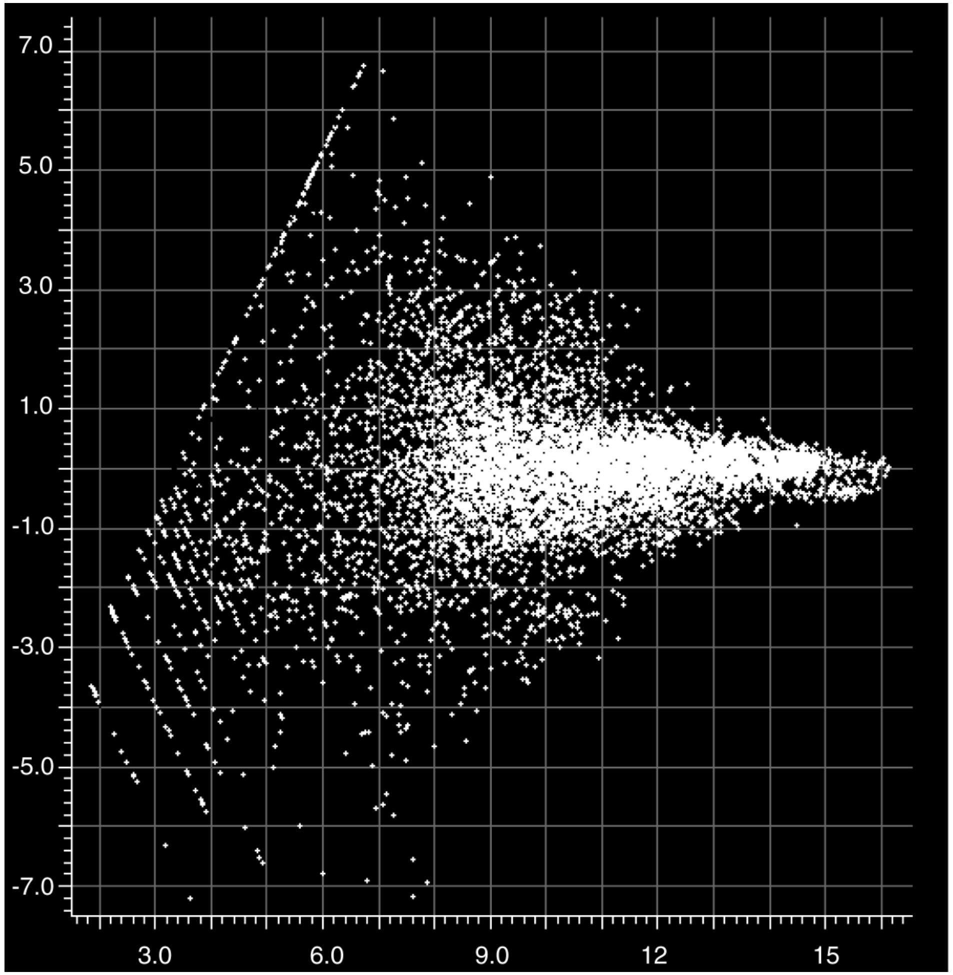

Normalised data (80 mg IB l^{-1} replicate 4)

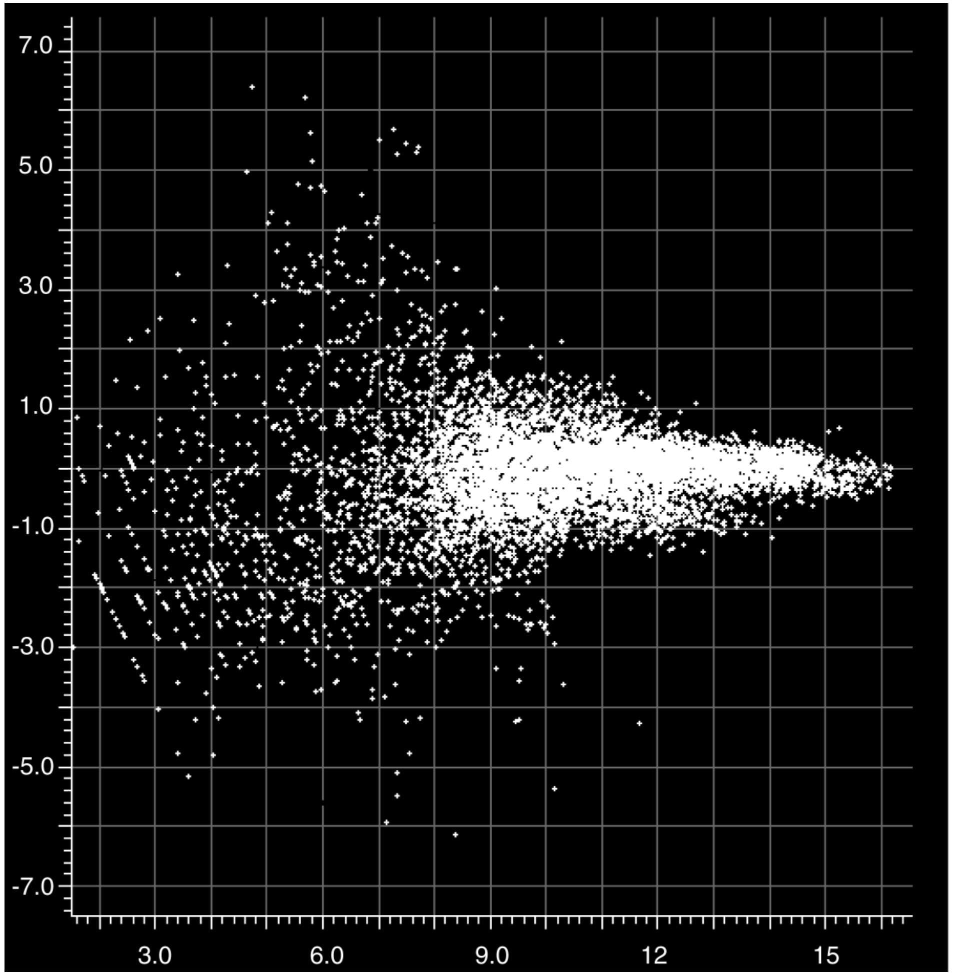

M (ratios)

A (intensities)

*Data were normalised using per spot and per chip intensity-dependent (Global LOWESS) normalisation followed by a per gene normalisation to the control samples, within each hybridisation batch, to normalise for batch variations.
