# Supplementary material for: Systems biology meets stress ecology: linking molecular and organismal stress responses in Daphnia magna
Source: Genome Biol. 2008 Feb 21;9(2):R40. doi: 10.1186/gb-2008-9-2-r40 (PMC2374704; doi:10.1186/gb-2008-9-2-r40)
Supplement: Additional data file 7 — Presented is a table showing water chemical parameters measured during the follow-up experiments. [file gb-2008-9-2-r40-S7.pdf]

Additional data file 7. Water chemistry of follow-up studies (mean ± SE).

	Adult exposure (n = 5)		Post-exposure rearing of neonates (n = 1)	Temporal exposure (n = 5)*
	**Day 4**	**Day 8**	**Culture media record**	**2-48 h**
**Conductivity (μS cm^{-1})**				
Control	400±4[a]	409±4[a]	393	445±7[a]
20 mg IB l^{-1}	408±4[a]	418±4[a]		
40 mg IB l^{-1}	415±5[a]	425±5[a]		
80 mg IB l^{-1}	424±4[b]	432±4[b]		462±5[a]
**pH**				
Control	8.12±0.02[a]	7.79±0.01[a]	7.22	7.66±0.02[a]
20 mg IB l^{-1}	8.22±0.02[ab]	7.78±0.01[a]		
40 mg IB l^{-1}	8.29±0.04[bc]	7.79±0.02[a]		
80 mg IB l^{-1}	8.43±0.05[c]	7.91±0.02[b]		7.69±0.02[a]

*Measurements are based on temporal sampling (2, 4, 8, 24 and 48 h) of pooled control or exposed samples. Different letters within the same parameter and column signify a significant difference ($P < 0.05$, Student's t-test).
