# Supplementary material for: Systems biology meets stress ecology: linking molecular and organismal stress responses in Daphnia magna
Source: Genome Biol. 2008 Feb 21;9(2):R40. doi: 10.1186/gb-2008-9-2-r40 (PMC2374704; doi:10.1186/gb-2008-9-2-r40)
Supplement: Additional data file 8 — Presented is a table listing technical data on QPCR (for instance, primers and amplification efficiency) from the follow-up experiment assessing temporal expression of key genes responding to ibuprofen. [file gb-2008-9-2-r40-S8.pdf]

Additional data file 8. Technical data on real-time quantitative PCR.

Target gene	Accession#	Primers (5'-3')[a]	Amplicon size (bp)	Amplification efficiency (E)[b]
Lip	BJ930771	CTGTTTATTGGCGACATTGC<br>TAGCCGTTCATTCTCCAACC	95	0.920±0.041
Ltb4dh	DY242190	AACCTACACCGAGGGTTTCG<br>TCCAACATTAACGCCATTAAGC	70	0.926±0.012
CTP	EV243383	AGATTCCATACTCGGCAGGA<br>CTCTGTGCGTTCTGTGGCTA	86	0.926±0.020
COX	DY037294	CCACCTCGCAAATTGTCTTT<br>GTCCCACGATGGATTCAACT	77	1.037±0.071
Cht	Contig961[c]	CACTCATTTGGTCTACGCTTTCG<br>CGGCTGGATTGTTGGGTTT	70	0.956±0.056
DmagVTG1	AB252737	CTGGCAAATGGGAAATCAAC<br>CCCAGGTGTAAGCCAAACC	93	0.857±0.020
FABP3	EV243384	GGCAAAGTGATGGTCCAAAC<br>TTCACATGCATCTCGTCTCC	89	0.850±0.034
JHE	BJ932560	ATGGAGTTCTCAACGGAACG<br>ATCTCAGGTGTGGGCATTTC	100	0.995±0.053
RXR	DQ530508	CCGGTTGGAATGAACTCTTG<br>TGGATAACGAGGCCAGTAGC	91	1.064±0.076
VMO1	EV243385	TATTACGCGGTTCAGACGTG<br>GTTGTCCGCCTCACTACCAT	87	0.822±0.035
**Reference gene**				
Act	AJ292554	CCACACTGTCCCCATTTATGAA<br>CGCGACCAGCCAAATCC	71	0.999±0.081
GAPDH	AJ292555	GGCAAGCTAGTTGTCAATGG<br>TATTCAGCTCCAGCAGTTCC	89	0.995±0.054
UBC	BJ928996	TCACCTGCACTCACCATTTC<br>AATCTCCGGAACCAAAGGAT	90	1.088±0.022

[a]Upper and lower sequences represent forward and reverse primers, respectively; [b]Values are mean ± SD (N = 80). Gene name abbreviations as in Fig. 1; [c]Contig sequence for *Cht* is available on DaphniaBase [55].
